# Can trophic rewilding reduce the impact of fire in a more flammable world?

**DOI:** 10.1098/rstb.2017.0443

**Published:** 2018-10-22

**Authors:** Christopher N. Johnson, Lynda D. Prior, Sally Archibald, Helen M. Poulos, Andrew M. Barton, Grant J. Williamson, David M. J. S. Bowman

**Affiliations:** 1 School of Natural Sciences and Australian Research Council Centre of Excellence for Australian Biodiversity and Heritage, University of Tasmania, Private Bag 55, Hobart, Tasmania 7001, Australia; 2 School of Natural Sciences, University of Tasmania, Private Bag 55, Hobart, Tasmania 7001, Australia; 3 Centre for African Ecology, School of Animal, Plant and Environmental Sciences, University of the Witwatersrand, Private Bag, Johannesburg, South Africa; 4 College of the Environment, Wesleyan University, 284 High St., Middletown, CT 06459, USA; 5 Department of Biology, University of Maine at Farmington, 173 High Street, Preble Hall, Farmington, ME 04938, USA

**Keywords:** herbivory, megaherbivore, fire regime, plant–animal interactions, pyrogeography, ecosystem engineer

## Abstract

Large vertebrates affect fire regimes in several ways: by consuming plant matter that would otherwise accumulate as fuel; by controlling and varying the density of vegetation; and by engineering the soil and litter layer. These processes can regulate the frequency, intensity and extent of fire. The evidence for these effects is strongest in environments with intermediate rainfall, warm temperatures and graminoid-dominated ground vegetation. Probably, extinction of Quaternary megafauna triggered increased biomass burning in many such environments. Recent and continuing declines of large vertebrates are likely to be significant contributors to changes in fire regimes and vegetation that are currently being experienced in many parts of the world. To date, rewilding projects that aim to restore large herbivores have paid little attention to the value of large animals in moderating fire regimes. Rewilding potentially offers a powerful tool for managing the risks of wildfire and its impacts on natural and human values.

This article is part of the theme issue ‘Trophic rewilding: consequences for ecosystems under global change’.

## 1. Introduction

Trophic rewilding aims to use the power of consumers—typically, large-bodied vertebrates—to sustain biodiversity and restore resilience to ecosystems degraded by past extinctions and other forms of human disturbance [1]. Most rewilding projects to date have used the reintroduction of large herbivores—either wild species or livestock undergoing ‘de-domestication’—to reinstate ecosystem functions that were lost with past extinctions and continuing population declines of wild herbivores [1,2]. These projects have emphasized the direct effects of herbivores on vegetation. In Europe, for example, rewilding with large herbivores has often been motivated by the idea that in the past, large herbivores created landscape mosaics of open and wooded habitats, with higher diversity than the tall closed forests that developed in the absence of control by herbivory [3–6]. These projects make little reference to the potential that large herbivores might also control the risks and impacts of fire.

In many ecosystems fire is a natural recurrent disturbance that can promote habitat heterogeneity and maintain biodiversity through complex interactions with food webs [7]. However, severe fires are a growing threat to natural environments and people globally [8] as global climate change increases the occurrence of weather conditions that promote high fire-danger [9]. In addition, risks to people are increasing as human settlement encroaches on fire-prone landscapes [8], while in some places abandonment of traditional land management due to rural depopulation has led to more large-scale fires [10]. Management responses to the threat of wildfire consist mainly of strongly interventionist actions: fire suppression, and reduction of fuel loads by prescribed burning and mechanical treatments such as forest thinning. However, fire suppression often results in accumulation of fuel loads, while prescribed burning is risky because fires can escape, especially as fire seasons become longer and more extreme.

Here, we ask whether rewilding can contribute to reducing the risks and impacts of wildfire in fire-prone landscapes. The core of the paper is a systematic review of the evidence that vertebrates are able to control fire regimes and of the mechanisms involved. Having considered this evidence, we place it in a biogeographic framework to identify the biomes and climate conditions under which effects of vertebrates on fire regimes have been demonstrated. We conclude by translating this evidence to current opportunities for rewilding projects in parts of the world where natural environments and human communities face increasing threats from destructive wildfire.

## 2. How vertebrates control fire

Vertebrates can influence natural fire regimes in several ways. First, herbivores limit fuel quantity by consuming and recycling plant matter that would otherwise accumulate as litter, and by reducing the density of vegetation [3]. Second, differential consumption of plant growth forms can enforce changes in the composition of vegetation and thereby alter the type and arrangement of fuel. Third, herbivory can generate large-scale habitat heterogeneity, as a result of variation in herbivore activity in response to factors such as terrain and water availability [3,11], and this can mean that zones of low and high flammability are interspersed in arrangements that could impede the spread of landscape fires. Finally, herbivores and other animals may alter the abiotic environment in ways that affect flammability: by forming trails, dust-baths or leks, large animals create lines or patches of bare ground that can act as fire breaks, while some species forage by turning over or digging through the litter layer and surface soils, and in the process bury fine fuels and thus reduce fuel loads. In the sections below, we explore the evidence supporting these effects of herbivores on fuel and fire regimes, in the past and present.

## 3. The evidence

### (a) Palaeo-ecology: megafaunal extinction

Many of the effects of herbivores on ecosystems are likely to increase with body size. This is because large herbivores are typically bulk feeders on low-quality plant material and thus consume a greater proportion of structural plant tissue than do small herbivores [12]. Further, the relative invulnerability to predation of the very largest herbivores can mean their populations escape top–down control and are instead limited by food availability, and so have greater impacts on plants than smaller species regulated by predation [13,14]. Because of their great physical power, megaherbivores have especially strong impacts by trampling or battering vegetation and disturbing the soil surface.

During the late Quaternary the largest herbivores vanished from most of the world's habitable continents and large islands in a wave of size-selective extinctions that followed the global expansion of modern *Homo sapiens* [15,16]. The loss of mammoths, ground sloths, giant kangaroos and other megafauna presents us with a grand historical lesson on the ecological consequences of removing large herbivores from ecosystems [17]. Several recent studies have used spores of dung fungi such as *Sporormiella*, which are obligatorily associated with vertebrate herbivores and sporulate only on their dung [18], to test the hypothesis that one of the effects of megafaunal extinction was to trigger increased fire [19]. Dung fungi are useful for these studies because their spores accumulate in sediments along with charcoal particles and pollen grains, so they allow us to match changes in herbivore activity in past ecosystems with dynamics of fire and vegetation.

So far, studies at 14 sites have used dung fungi to track herbivore decline through time intervals spanning regional extinction of megafauna (and arrival of humans), while providing matching records of charcoal and pollen (see electronic supplementary material, table S1 for details). At six of these sites the vegetation was forest or woodland before megafaunal extinction, and at these sites extinction was associated with large increases in charcoal. In several of these cases the temporal resolution of sampling was fine enough to show that charcoal increase followed dung-fungus decline on time-scales of decades or centuries [20–24]. In some places, increased fire was followed in turn by changes in vegetation: from open mixed rainforest and sclerophyll forest to uniform sclerophyll forest at Lynch's Crater in NE Australia [20]; from patchy spruce parkland to continuous hardwood and conifer forest at Appleman Lake in the NE USA [22]; and from mosaics of savannah, woodland and thicket vegetation to extensive grassland in SW Madagascar [24,25].

In contrast, megafaunal extinction was associated with no apparent change in fire or vegetation at sites where the original vegetation was treeless tundra, steppe or arid grassland [26–31]. The variation in response is illustrated in electronic supplementary material, figure S1: synchronous herbivore declines in the far northeast and far southwest of the Australian continent were associated with a rise in charcoal in the forested NE, but with no change in the dry low shrubland habitat of the SW.

These are all observational studies that are subject to several uncertainties of interpretation. The most obvious is that we cannot test if increases in charcoal in past ecosystems truly signal higher flammability in response to relaxation of herbivory, or whether they had other causes such as rapid climate change or firing of the landscape by newly-arrived people (who may also have hunted the big animals to extinction). Also, charcoal concentrations in sediments are at best a crude measure of fire regimes [32]. For example, it is often difficult to distinguish frequency from intensity of fire using charcoal records. It is possible that in cases with no apparent change in charcoal there were still important shifts in fire regime, especially the spatial pattern of burning.

Nonetheless, the evidence to date supports the hypothesis that past disappearances of large herbivores triggered increased fire severity or frequency, at least in places where abiotic conditions allowed development of woody vegetation. In such environments, increases in plant biomass due to relaxation of herbivore pressure may have been large enough to shift landscapes to states in which fire was more frequent or more intense, or both. In less productive environments, either too cool or too dry for development of woody vegetation cover—especially under the low-CO_2_ conditions of the last glacial cycle—increases in fuel following loss of large herbivores may have been too small to cause observable changes in charcoal accumulation.

### (b) Extant herbivores

There is overwhelming evidence that extant large herbivores reduce herbaceous fuel loads. For example, grazer exclusion in Hluhluwe iMfolozi Park in South Africa led to increased grass biomass [33]; in mixed-conifer forests in the NW USA, understorey biomass was higher inside than outside exclosures for ruminants [34]; and a meta-analysis of 7615 records from mostly semi-arid and arid ecosystems of Australia under natural field conditions found that livestock reduced plant biomass by an average of 40% [35]. The direct effects of herbivory on fuel are often clearer for grazers than browsers because grazers consume a larger proportion of the individual plants they eat than do browsers [36]. Because the literature on herbivory and fuel loads is vast we have not reviewed it systematically; instead we indicate the main effects, with supporting studies, in electronic supplementary material, table S2.

While there are many studies demonstrating that vertebrate herbivores reduce fuel accumulation, there are relatively few which demonstrate that these reductions of fuel are sufficient to affect fire regimes. The available evidence is summarized in table 1. Compelling observational studies include the Ithala Game Reserve in South Africa, where 64 years of aerial photography and 30 years of field measurements showed that herbivore populations were inversely related to accumulation of grassy biomass and therefore the likelihood of fire [59]. Several African studies show that concentrations of grazers produce lawns in which fire is rare [43,45,46,60,61], and similar ‘marsupial lawns’ occur in Tasmania [58]. At large scales, there is a negative relationship between grazer biomass and fire frequency in African savannahs [43,51,62]. In the steppes of southern Russia, declining livestock populations since the fall of the Soviet Union in 1991 was followed by rapid increase in the area burned by wildfire, evidently because of increased fuel loads [49].

**Table 1.** Studies of the effects on fire regimes of terrestrial vertebrate herbivores, including native wildlife and domestic livestock in intact (uncleared) landscapes. Effects due to *grazers* (species that feed predominantly on grass, often including other graminoid or herbaceous plants) are distinguished from effects due to *browsers* (species that feed predominantly on woody plants). Evidence types are manipulative experiments (E), modelling of relevant data (M), and correlational or observational (C). ‘Strength’ of evidence is rated on a 3-point scale (3 is strongest), as judged by a combination of effect size, type of studies, number of studies and diversity of environments in which the effect has been demonstrated, as well as existence of a plausible underlying mechanism.

effect	evidence:		refs (listed in the electronic supplementary material)
	type	strength	
Less biomass consumed by fire in *grazed* areas	E, M	3	[36–38]
Fire temperatures and flame height are lowered because of reduction of fuel loads by *grazers* (and possibly *browsers*)	E, M	2	[36,39,40]
Fire-induced mortality of sensitive plants is reduced in *grazed* areas	E	2	[37,41]
Fire severity is possibly increased in areas *grazed* by cattle, because of increased fuel loads from unpalatable shrubs	C	2	[42]
Rate of fire spread is reduced because of reduction of fuel loads by *grazers* (and possibly *browsers*)	E, M	2	[39,40]
Area of landscape burned is reduced because short-grass patches created by *grazers* (‘grazing lawns’) impede the spread of fires	E, C, M	2	[43–46]
Area burned is reduced because *grazing* lowers fuel loads and breaks fuel continuity	M, C	2	[38,47–50]
Return interval of fire is lengthened because of increased woody cover and smaller herbaceous fuel loads due to *grazing*	M, C	2	[51–57]
Number of potential fire days is reduced because of reduced fuel loads due to *grazing*	M	1	[58]
Number of potential fire days may be increased in tussock grassland because *grazers* selectively remove live shoots, increasing the proportion of dry dead fuels	M	1	[58]

Experimental studies showing effects of vertebrate herbivores on fire include Kimuyu *et al.* [39], who show that in a Kenyan savannah, plots grazed by wildlife and cattle experienced lower burn temperatures than ungrazed plots, due to lower herbaceous fuel loads. Likewise, experimental manipulation of grazing in tallgrass prairie in Kansas demonstrated that grazing reduced fire temperatures and energy release [36]. In grasslands of Yellowstone National Park, sites occupied by elk (*Cervus canadensis*) had a sixth of the litter and standing dead biomass compared to unoccupied sites, and fire on those sites consumed less of these fuels and caused a smaller increase in bare ground [63]. A long-term experiment in sagebrush (*Artemisia*) communities in Oregon found that consumption of fine fuel by fire was lower with moderate intensity pre-fire cattle grazing compared to ungrazed exclosures, resulting in reduced mortality of large perennial bunchgrasses [37]. A large-scale experiment in tropical Australia found that, in the absence of non-native swamp buffaloes (*Bubalus bubalis*), mortality of juvenile trees was three times higher in burnt than unburnt plots, but in the presence of buffaloes there was no difference in mortality between burnt and unburnt plots [41].

Several different modelling approaches have been applied to the relationship of vertebrate herbivores and fire regimes. One class of studies uses modelled relationships between fuel loads and fire behaviour to predict changes in fire resulting from herbivore-caused alterations of fuel. In high-altitude Ethiopian *Erica* shrublands, cattle grazing resulted in slower post-fire fuel accumulation and discontinuous litter, and modelling suggested this would reduce fire intensity and rate of spread [64]. In the Serengeti ecosystem, fire models predict that at low grazing intensity, high grass biomass leads to extensive fires [38], while a continental-scale model of African vegetation predicted large reductions of grass biomass, and therefore area burned, due to grazers [47]. The spatial distribution of fuel, as well as its average quantity, are important. For example, fire models suggest that herbivores can alter fire spread by changing the size of fuel patches [44]. Other approaches take models of vegetation dynamics and extend them to include the interacting effects of fire and herbivory on the development of vegetation structure and composition [65]. New dynamic global vegetation models (DGVMs) are being developed that incorporate trait values for individual plants and depict competition among plants more realistically than earlier DGVMs that model fixed growth forms; these models are also capable of describing complex and recursive interactions between fire and the structure and composition of vegetation [66], and could potentially be extended to add interactions with herbivory as well [67].

### (c) Grazers versus browsers

The strongest evidence that vertebrate herbivores affect fuel loads and fire regimes comes from studies of large grazers, rather than browsers (table 1; electronic supplementary material, table S1). When grass is abundant, it provides a dense and continuous layer of material that under dry conditions turns quickly into a flammable bed of fuel. Fires often start in the grass layer, where they gain intensity and ultimately consume woody tissue when flame height is sufficient to reach the canopies of shrubs and small trees. The flammability of grasses can sustain grass-fire cycles [68] in which rapid seasonal replenishment of grass fuel supports recurrent fire with short return times that kill woody regeneration. However, grass biomass responds quickly to changes in grazing, and even quite patchy grazing can decrease fuel continuity in grassland sufficiently to reduce fire frequency, extent and intensity [46,69]. Large grazers can also maintain spatial variability in plant-community composition through the process of pyric herbivory, in which grazing moderates but does not eliminate fire when grazers concentrate on recently burned areas, thus allowing some accumulation of fuel elsewhere and sustaining a fire mosaic that prevents general encroachment of woody plants [68]. The effects of browsers on fire regimes are often more complex. In Africa, large browsers open up closed canopies and so allow grasses and fire to penetrate wooded landscapes, while browsers of all sizes promote high loads of herbaceous fuel by preventing recruitment of trees [62,70]. Large browsers can reduce vegetation density and create open habitats, but whether this reduces fire frequency or impact can depend strongly on the complementary effects of grazers—or mixed feeders—in controlling grass fuels.

Grazing does not always lead to reduced fire. In the Australian Alps, cattle grazing evidently increases fire severity, possibly by changing fuel arrays in favour of flammable woody shrubs that can encroach on grasslands [42]. In situations where woody plants dominate fuels, browsers might control fire regimes. For example, in Mediterranean oak woodland in Israel, browsing by cattle can reduce cover and biomass of shrubs and trees and thereby limit accumulation of flammable plant material [71]. Unfortunately, there remains a lack of evidence on the effects of browsing on fire regimes in wooded landscapes.

### (d) Ecosystem engineers

Scattered anecdotal observations suggest that animal trails and similar disturbances can function as firebreaks. The physical scale of these features can be substantial. For example, in a montane vegetation complex in Tasmania, 10% or more of the ground surface was covered by animal paths, mostly created by medium-sized macropods and wombats. Path surfaces were either bare ground or compressed leaf litter [72]. The effects of path networks on fire behaviour could well be significant, particularly by impeding the spread of low-intensity fire, but this appears not to have been systematically studied.

Vertebrates that dig for their food or otherwise disturb the litter layer can also affect fuel by burying plant litter or mixing it with soil. Some of these engineers have large physical effects on soil and litter. For example, in Switzerland, grubbing by wild boar (*Sus scrofa*) may disturb 27–54% of the forest floor [73]. How such activities affect fire is mostly unknown, other than from a recent series of studies in Australia. A study of the woylie (*Bettongia penicillata*), a marsupial that digs for truffles and invertebrates, found that its activity reduced surface litter loads in dry woodlands by 25%; this translated to a 74% reduction in flame height and a 33% reduction in the rate of fire spread predicted by a fire model [74]. Southern brown bandicoots (*Isoodon obesulus*) had a similar effect on fire predictions in remnant woodland in the city of Perth [75]. Superb lyrebirds (*Menura novaehollandiae*) forage by turning over surface soil, burying leaf letter in the process. This activity reduced litter loads by 25% over nine months in plots where lyrebirds were free to forage, compared with plots from which they were excluded. Fire models predicted that this would cause up to twofold reduction of flame height, depending on weather [76]. Mound-building by malleefowl (*Leipoa ocellata*) reduced fuel loads and model-predicted fire intensity in the vicinity of mounds [77].

## 4. A biogeographic synthesis

**Figure 1.**
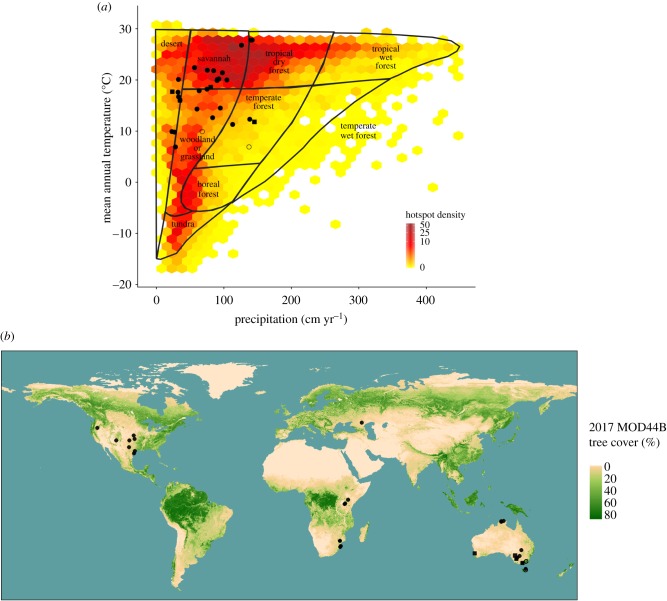
Locations of studies of effects of vertebrates on fire regimes. These are shown in relation to (*a*) global variation in mean annual temperature and mean annual rainfall, with biomes superimposed (from [78]), and relative density of fires (hotspot density) as detected at 1 km resolution by the satellite-based Moderate Resolution Imaging Spectroradiometer (MODIS) instrument; and (*b*) geography, with variation in tree cover also shown. Filled circles are studies demonstrating reduction of fire activity (frequency, intensity or extent) due to herbivores; open circles are studies demonstrating no reduction of fire due to herbivores; and filled squares are studies showing reduction of fire activity by ecosystem engineers. The studies involving herbivory are those listed in table 1 that were conducted at specific localized sites; studies of ecosystem engineers are described in the text. Palaeoecological studies are not included.

In figure 1 we summarize the environmental distribution of studies that have tested the effects of vertebrates on fire regimes, against the background of Whittaker's [78] global ordination of the occurrence of biomes in relation to temperature and rainfall. This shows that evidence for limitation of fire by vertebrates is concentrated in the world's savannahs, woodlands and grasslands, in a region of environmental space with intermediate temperature and rainfall, where current fire activity is also high (figure 1*a*).

The geographical pattern of evidence for herbivore control of fire resembles a well-established pyrogeographic pattern of effects of fire on vegetation [79], in which such effects are strongest at intermediate rainfall and temperature. In arid environments, plant biomass is generally too sparse to support frequent fire, while in wet environments flammability is too low. At intermediate rainfall, fuel can reach levels of both biomass and flammability that result in recurrent fire, provided plant growth is not limited by temperature. This relationship gives rise to a large environmental and spatial domain within which the state of vegetation—whether grassland, woodland or savannah—is not fully determined by climate but is strongly influenced by biomass removal by fire [80–83]. Where fire is a recurrent disturbance within this domain, trees remain sparse and flammable grasses often dominate; reduction of fire allows development of tree cover, which then drives reduction of grass fuel.

By removing fuel, large herbivores can to some extent replace fire as a dominant control of vegetation structure and density. This replacement is best understood in African savannahs. In savannahs with mean annual rainfall between approximately 500 and 800 mm, either fire or herbivory may be the dominant control on vegetation biomass [62]. Notably, it is rare to find sites with intermediate consumption by both herbivores and fire in this environmental domain, which contains many of Africa's most important conservation areas: similar ecosystems contain either many herbivores or are extensively burned [62]. In replacing fire, large herbivores like white rhinoceros (*Ceratotherium simum*) create habitat heterogeneity and facilitate many other species (see Case Study 1 in the electronic supplementary material).

Geographically, studies of the effects of vertebrates on fire are concentrated in Africa, North America and Australia (table 1 and figure 1*b*). More research is needed in environments such as Mediterranean woodlands and shrublands and temperate forests worldwide.

## 5. Implications for rewilding

**Figure 2.**
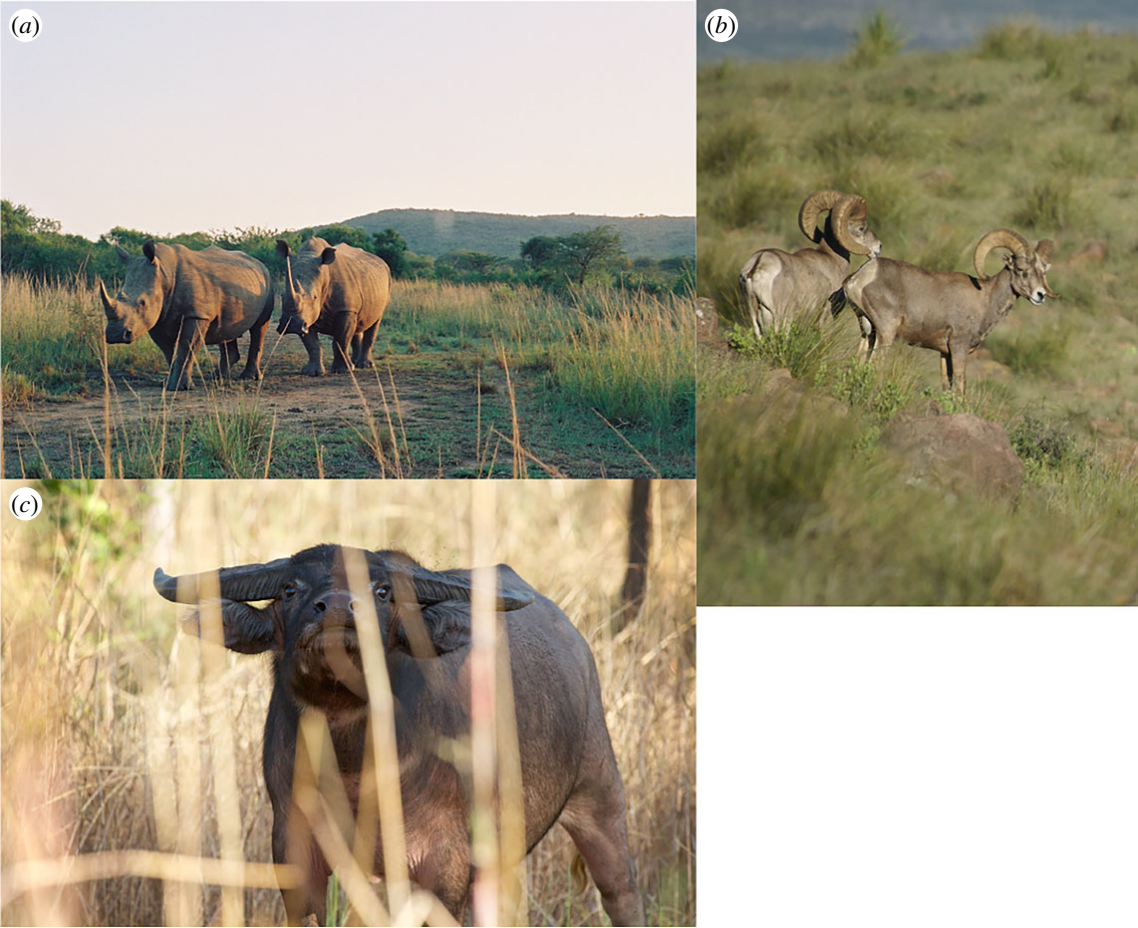
The potential, and complexity, of trophic rewilding for management of fire regimes as illustrated by three case studies detailed in the electronic supplementary material: (*a*) white rhinos and other large herbivores control fire in conservation reserves in southern Africa (image: Sally Archibald); (*b*) rewilding of communities of large herbivores may reduce the threat of wildfire in the southwestern USA (image: Louis Harveson); and (*c*) the introduced swamp buffalo may be an ecological replacement for extinct Pleistocene megafauna in northern Australia with ecological benefits that must be traded off against unwanted impacts (image: David Hancock).

While trophic rewilding is potentially a powerful tool in the management of fire, its implementation will depend on many social, historical and environmental factors specific to particular landscapes. We illustrate some of these variations using detailed case studies of management of changing herbivore populations in relation to fire dynamics (figure 2), detailed in the electronic supplementary material. They are: *in situ* recovery of native large herbivores (white rhinos and other grazers) from low levels enforced by past removals, and its role in management of conservation areas in southern Africa; intentional restoration of native large-herbivore communities in high-elevation woodlands in southwestern North America, where vulnerability to fire has recently increased due to increased fuel loads resulting from decline of domestic livestock, as well as climate change; and the effects on fire regimes of an invasive large herbivore, the swamp buffalo (*Bubalus bubalis*) in northern Australia that may be a partial ecological replacer for extinct megafauna, but with unwanted environmental impacts as well as beneficial effects on fire regimes.

Rewilding for management of fire could be especially important where recent changes in land use have increased the risk of dangerous wildfire. In much of the Mediterranean region, for example, original populations of large herbivores have long disappeared, but until recently fuel loads in managed savannah environments were controlled by domestic livestock [10,71]. With recent farm abandonment, and replacement of traditional management by activities such as commercial forestry, the incidence of destructive fire has increased [84]. Rewilding may be an option for the future use of abandoned landscapes [85]. If this option is to be viable it may be essential that it includes re-establishment of large herbivores to control risks of wildfire. As noted above, more research is needed on the effects of large herbivores at natural densities on fire regimes in such environments.

Large grazers may be especially important agents of fire control, given that they evidently have strong and consistent effects in reducing fuel loads and moderating the extent and impacts of fire in highly fire-prone environments such as savannahs. Grazers accomplish general reductions of fuel loads in biomes with a significant grass component, and they may also create and maintain open spaces with low fuel loads—grazing lawns—that can impede fire spread. Potentially, grazing lawns could be initiated by managers using mechanical removal of woody cover to stimulate localized grass growth and recurrent grazing. This has been attempted in the Kruger National Park, in response to concern that frequent large fires were creating undesirable habitat and limiting herbivore numbers [45]. Ironically, fire was used as the tool to re-set this system. Short-grass ‘grazing lawns’ were created in a fire-prone tall-grass system by burning small patches; these attracted and concentrated native wildebeest and other herbivores; intense grazing produced short-grass lawns that subsequently did not burn [45].

In other situations, designed grazing lawns could be used to break up otherwise contiguous flammable vegetation, such as *Pinus* and *Eucalyptus* forests, in strategic configurations likely to be effective in containing fires and protecting sensitive environments or human communities. Browsers could be valuable in reducing woody vegetation cover and creating habitat mosaics, but at the potential cost of increased grass and raised fire-danger; they should be complemented by grazers to prevent this. On the other hand, heavy grazing can lead to shrub encroachment [68], and in some cases dense shrublands can also be highly flammable [86]. Mixed assemblages formed by introductions of both grazers and browsers may well be most beneficial in developing stable landscape mosaics with low or moderate fire-danger. Large-bodied mixed feeders capable of consuming large quantities of grass, such as the European bison (*Bison bonasus*) [87,88], could be especially valuable in controlling fire while creating open and patchy habitats [89].

The non-consumptive effects of ecosystem engineers on fuel loads may well be a valuable tool in rewilding for management of fire. This is especially true in Australia, because many of the species that are most active in digging for food and mixing litter with top soil are medium-sized marsupials and large rodents that have recently undergone severe declines caused by invasive predators [90]. Some of these species persist in island refuges and there is potential for their large-scale reintroduction to mainland ecosystems, provided that impacts of predators can be reduced sufficiently. Restoration of digging mammals could play a role in reducing fire-risk, while also renewing other ecological services such as improving soil condition and promoting regeneration of some plants [91].

## 6. Conclusion

Our review of the evidence makes it clear that vertebrates can have strong effects on fire regimes. Increases in fire frequency, severity and extent due to decline of large vertebrates probably began with extinction of Quaternary megafauna. Recent and continuing declines of large herbivores could well be contributing to increases in wildfire now being experienced in many parts of the world [8], including places where catastrophic fire threatens environmental values and human communities [10]. However, our knowledge of the effects of vertebrates on fire is still sparse, as well as being unevenly distributed in geographical and environmental space. While many studies document effects of herbivores on fuel loads, and often infer that those changes are likely to moderate fire regimes, surprisingly little research has gone on to test whether those effects cause significant changes in the frequency, extent and severity of fire, and that research is concentrated in savannah and grassland environments. We still know little about how the effects of herbivores and other ecosystem engineers vary among species and habitats, and how they are related to the density and behaviour of animals. Further, while our conception of trophic rewilding includes feral and domesticated animals as well as native species, it is not clear to what extent the available diversity of domesticated herbivores is able to emulate the spectrum of effects on fire regimes that can be achieved by non-domesticated species, including surviving megafauna. Research on these questions is needed for the application of trophic rewilding as an effective tool in controlling fire.

## Supplementary Material

Supplementary Material

## Data accessibility

This article has no additional data.
