# Supplementary material for: Can trophic rewilding reduce the impact of fire in a more flammable world?
Source: Philos Trans R Soc Lond B Biol Sci. 2018 Oct 22;373(1761):20170443. doi: 10.1098/rstb.2017.0443 (PMC6231065; doi:10.1098/rstb.2017.0443)
Supplement: Supplementary Material [file rstb20170443supp1.docx]

**Supplementary Information**

**A. Paleoecological studies of temporal change in dung fungi, charcoal and pollen: Table S1**

**B. Summary of studies on effects of large herbivores on fuel loads, fuel type and arrangement, and on fire regimes: Table S2**

**C. Process for Systematic Review of Effects of contemporary large herbivores on fire regimes**

**D. Case study 1: Rewilding the native herbivores of Southwestern North America**

**E. Case study 2: Asian Water Buffalo (*Bubalus bubalis*) in northern Australia**

**F. Case study 3: White rhino in Southern Africa**

**A. paleoecological studies of temporal change in dung fungi, charcoal and pollen**

Table S1. Paleoecological reconstructions from sedimentary records, at sites where large-herbivore decline is indicated by falls in spores of dung fungi coinciding with regional megafaunal extinction, and with matching records of change in fire (from charcoal) and vegetation (from pollen). Date of spore decline is shown as thousands of years (ka) before present, based on calibrated radiocarbon chronologies provided in the source papers. On Santa Cruz, dung-fungus decline reflects extinction of tortoises from the high-elevation interior, though they persisted on the coast. Studies are tabulated in order of age of dung-fungus decline and distinguished according to whether (green shading) or not increased charcoal concentrations were associated with dung-fungus decline.

Notes on Table S1:

This compilation is restricted to studies that use the dung-fungus proxy to indicate changes in populations of herbivores, because dung fungi provides records that can be aligned with records of fire and vegetation, from charcoal and pollen recovered from the same sediments. The justification for treating spores of dung fungi as a proxy for herbivores, especially large herbivores, is provided in refs [1, 2]. Several recent studies have validated this proxy as an indicator of the abundance of living large herbivores [3-5]. In several cases, for example the South Island of New Zealand [5] and the sub-Antarctic Enderby island [6], spores of dung fungi faithfully represented the introduction and later decline or extirpation of large herbivores. The dung-fungus proxy has also been validated as in indicator of megaherbivore extinction, in the case of the woolly mammoths of St Paul Island, where *Sporormiella* fixed a date (5,600 years ago) on extinction that matched almost exactly the timing of extinction determined from environmental DNA and from a well dated collection of fossils [7].

Two studies were not included in the compilation in Table S1. Raczka et al. (2017*)* used *Sporormiella* to date decline of large herbivores to 12-11 ka at two sites in southeast Brazil [8]. Although they report that charcoal increased in the early Holocene, it is not clear whether isolated charcoal peaks around 12-10 ka might be associated with slightly earlier *Sporormiella* decline. A study on the South Island of New Zealand described *Sporormiella* declines attributed to extinction of moa and other large flightless birds. This study did not report on charcoal. Other studies have shown that fire was rare before human arrival in much of New Zealand because of absence of sources of ignition, but increased very soon after almost certainly because of purposeful burning by people [9].

The study reporting *Sporormiella* and charcoal in southwest Madagascar [10] also detailed several other sites where *Sporormiella* counts were consistently low, so these are not included.

| **Location** | **Date of spore decline** | **Pre-decline vegetation** | **Change** | **source** |
| --- | --- | --- | --- | --- |
| South West Western Australia | 43 ka | Dry shrub & grassland | No change in vegetation or charcoal | [11] |
| NE Australia, Lynchs Crater | 42 ka | Mixed wet & dry rainforest & sclerophyll forest | Transition to grassy sclerophyll (*Eucalyptus*) forest | [12] |
| SE Australia, Caledonia Fen | ~50-40 ka | Grass/shrub steppe | No change in vegetation or charcoal | [13] |
| Peru, Lake Pacucha | 15.8 ka | alpine/subalpine grassland | Dung-fungus decline correlated with increased woody taxa, increased charcoal | [14] |
| Silver Lake, ohio USA | 13.9ka | Spruce parkland | Charcoal increase followed closely on dung-fungus decline; vegetation shifted to mixed hardwood/conifer forest | [15] |
| Applemen Lake, Indiana USA | 13.7 ka | Spruce parkland | Charcoal increase followed closely on dung-fungus decline; vegetation shifted to mixed hardwood/conifer forest | [16] |
| Lough Nadourcan | ~13.5 ka | Low *Empetrum* heathland | No increase in charcoal with dung-fungus decline. Vegetation remained *Empetrum* heath, until increase in *Betula* with climate warming around 11 ka. | [17] |
| Long Lough, Ireland | ~13 ka | Steppe tundra | No increase in charcoal with dung-fungus decline; no vegetation change | *Jeffers et al 2018* |
| New York State, Binnewater Pond | ~13ka | Pine & spruce forest | Charcoal increase follows dung-fungus decline; conifer forest persists, with increased grass | [18] |
| Florida, Page-Ladson site | 12.7 ka | Mesic hardwood forest or ‘parkland’ | Dung-fungus decline associated with increasing grass and charcoal, and transition to *Pinus* woodland. | [19, 20] |
| Quidenham Mere, Norfolk, England | 12.2 ka | Steppe-tundra | Charcoal decline soon after drop in dung-fungus; subsequent development of oak woodland linked to Holocene warming | [21] |
| St Paul Island, Alaska | 5.6 ka | Tundra | No vegetation change; charcoal absent before and after mammoth extinction | [7], J. W. Williams *pers. comm.* |
| SW Madagascar, Ambolisatra | 1.72 ka | Mosaic of woodland, savanna, and thicket | Charcoal increase follows dung-fungus decline, expansion of open grasslands | [10, 22] |
| Santa Cruz Is, Galapagos | 500-700 | Freshwater wetlands, plus..? | Charcoal increase coincided with dung-fungus decline; wetlands replaced by sphagnum bogs | [23] |

**B Table S2. Studies relating large herbivores to fuel loads, fuel type & arrangement, and fire regimes.**

Studies are rated according to type and strength of evidence. Vertebrate effects on fuels and fire, according to evidence type and strength. The studies are of native wildlife and domestic livestock in relatively intact (uncleared) landscapes. References regarding fuel load are a representative sample rather than exhaustive, given the plethora of studies showing an effect. Evidence type (from strongest to weakest): A – meta-analysis of multiple datasets; E: manipulative experiment; M – modelling of relevant data; C –correlative (e.g. from natural gradients); and O – observational. ‘Strength’ is a subjective assessment of the strength of evidence, based on the effect size, type of studies, number of studies, and diversity of environments in which the effect has been demonstrated, as well as existence of a plausible underlying mechanism.

|  | Effect | Evidence: | | Refs |
| --- | --- | --- | --- | --- |
|  |  | type | strength |  |
| ***a. Fuel loads*** |  |  |  |  |
| Litter fuels | **Grazers** reduce mass and cover of leaf litter & fine woody debris | A, E | 3 | [24-27] |
| Grass and herbaceous fuels | **Grazers** reduce biomass, height, cover, continuity and accumulation rates | A, C, E, M | 4 | [24, 28-35] and others |
|  | Large **browsers** can increase herbaceous biomass by preventing tree recruitment | O | 1 | [36] |
| Understorey | **Elephants** can increase understorey plant biomass by creating ‘tree guards’ from damaged canopies | E | 1 | [37] |
| Shrub fuels | **Herbivores** reduce leaf biomass, growth rate and continuity | E | 3 | [38-41] |
| Elevated (canopy) fuels | Large mammalian **browsers** reduce tree density and biomass and slow recovery after fire by consuming woody sprouts | E, C, M | 3 | [32, 34, 36, 42-46] |
|  | **Grazers** can increase woody vegetation through reducing grass competition | E, C | 2 | [27, 47-49] |
| ***b. Fuel type and arrangement*** | | | | |
|  | **Grazers** can increase woody fraction (in some cases, converting grasslands to shrublands) | A, C | 3 | [47, 50] |
|  | **Elephants** can open up closed canopies and allow grasses and fires to penetrate | O | 1 | [51] |
|  | **Herbivores** can change woody plant composition (e.g. decrease palatable deciduous trees, increase unpalatable/flammable conifers that serve as ladder fuels) | E, C, M | 3 | [43, 47, 52, 53] |
|  | **Grazers** can create and maintain grazing lawns | E, C | 3 | [54-56] |
|  | **Grazers** can alter heterogeneity and grain size of patches in the landscape | E, M | 3 | [57-59] |
|  | **Grazers** can increase flammability of post-fire plant species mix (some opposing effects) | E | 1 | [60] |
| ***c. Fire regimes*** |  |  |  |  |
|  | Less biomass consumed by fire in **grazed** areas (and presumably less energy released by fire) | E, M | 3 | [61-63] |
|  | Lower fuel loads due to **grazers** (and possibly **browsers**) reduce fire temperatures and flame height | E, M | 2 | [30, 31, 62] |
|  | Less fire-induced mortality of sensitive plants in **grazed** areas | E | 2 | [27, 61] |
|  | BUT **grazing** does not always lead to reduced fire severity; cattle grazing can possibly increase fire severity by changing fuel arrays | C | 2 | [64] |
|  | The smaller fuel loads due to **grazers** (and possibly **browsers**) slow rate of spread | E, M | 2 | [30, 31] |
|  | Grazing lawns and the altered grain size of patches and created by **grazers** can reduce area burnt in a landscape | E, C, M | 2 | [54, 65-67] |
|  | **Grazing** reduces fuel loads and fuel continuity, leads to less area burned | M, C | 2 | [34, 63, 68-70] |
|  | The increased woody cover and smaller herbaceous fuel loads due to **grazing** are associated with a longer fire return interval | M, C | 2 | [71-77] |
|  | Although **grazing** typically reduces the number of potential fire days by reducing grass fuel loads, in tussock grasslands it can increase potential fire days by increasing proportion of dry dead fuels | M | 1 | [78] |

**C. Process for Systematic Review of Effects of contemporary large herbivores on fire regimes**

We used a systematic review for the effects of herbivores on fire. We searched the Science Citation Index Expanded (SCI-EXPANDED) from 1945 to the present, and the Conference Proceedings Citation Index-Science (CPCI-S) from 1990 to the present. We applied three sets of search terms in sequence, as shown below:

| Set | Terms | Number of references |
| --- | --- | --- |
| A | <herbivor*> OR <folivor*> OR <grazer> OR <browser> | 57,929 |
| B | <fire> | 180,495 |
| C | <A> AND <fire> | 1466 |

We then examined all the references in set C and removed studies of invertebrates; all remaining studies were of mammalian herbivores. We then selected studies of effects of herbivores on fire in relatively undisturbed ecosystems, without extensive clearing of conversion of original native vegetation. We did not apply a body-size criterion to represent large herbivores, but the species that remained after applying the filters ranged in size from dik-dik (~ 4 kg) up to elephant. These studies were then placed into three classes according to evidence type: experimental (i.e. manipulative experiments, usually involving grazing exclosures); correlative (using natural gradients and contrasts of site with differing herbivore density) or modelling (modelling fire behaviour as predicted from measured fuel loads under different herbivore regimes). A 3-point rating of strength of evidence (3 is strongest) was applied to each study. This was judged on a combination of sample size, evidence type (with manipulative studies rated as strongest), effect size and a clear underpinning mechanism

**D. Case study 1: Rewilding the native herbivores of Southwestern North America**

**Summary**

- During the Pleistocene the American Southwest supported a rich megaherbivore fauna. This fauna was mostly extinct by 11,500 YBP, probably because of Paleoindian hunting.
- Despite these extinctions, populations of some large herbivores, such as pronghorn antelope, desert bighorn sheep, white-tailed deer, and mule deer, continued to graze and browse the vegetation of the region.
- Widespread Euro-American settlement in the late 1800s led to drastic decline in these native herbivores through hunting and replacement by livestock, especially cattle.
- These changes in combination with active fire suppression led to a dramatic reduction in naturally-frequent, low-intensity surface fires in the 20^th^ century, and a consequent build-up in fuels.
- On both sides of the US-Mexico border, private and public land owners are rewilding lands with native megaherbivores that were common before settlement. The goals of these initiatives are to re-establish native species, enhance livelihoods of local people, and restore ecosystem function.
- These efforts may have the added benefit of reducing fuel and mitigating the risk of dangerous, uncharacteristic wildfire.


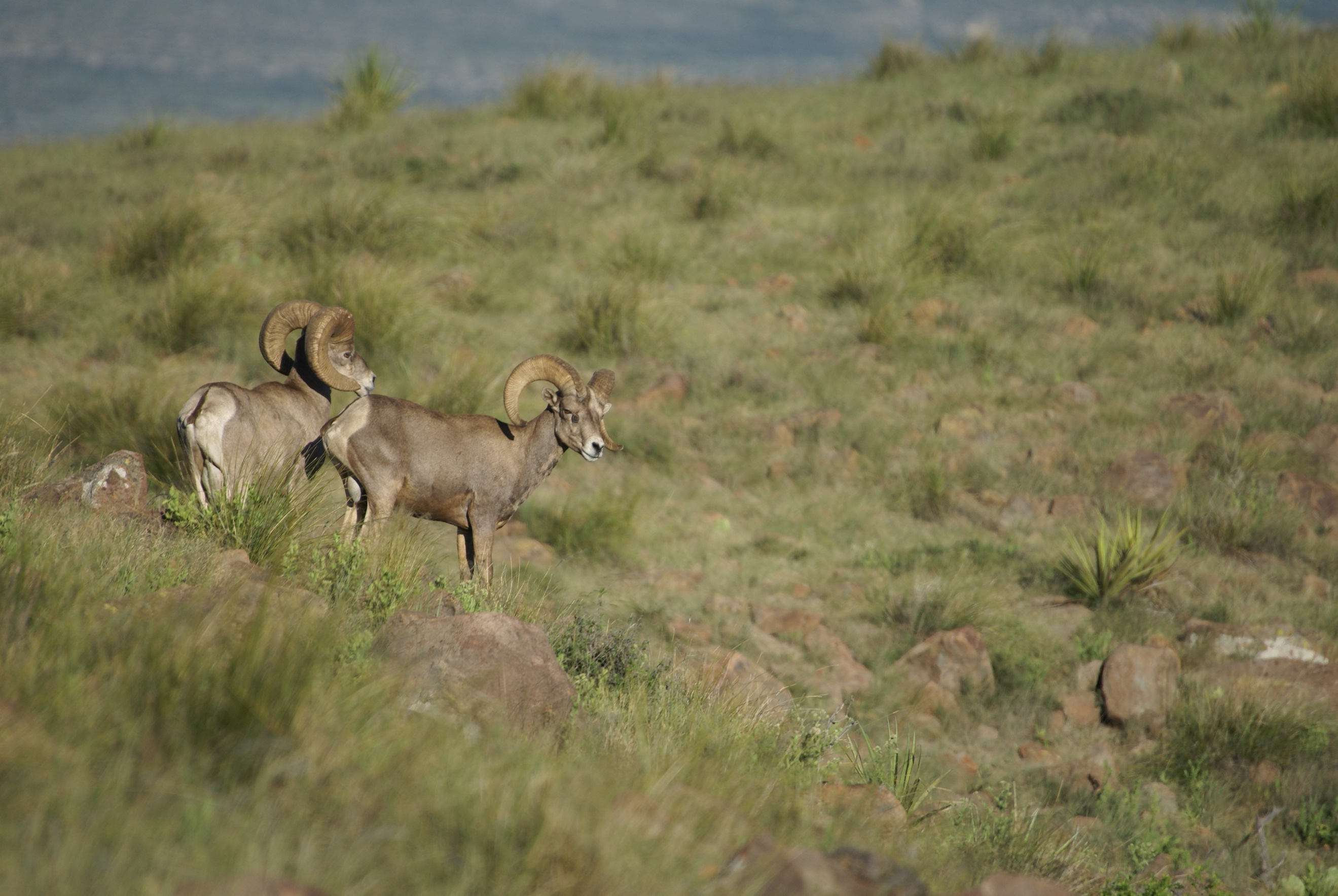


Figure S1. Desert bighorn sheep *Ovis canadensis nelson* in the American southwest (photo credit: Louis Harveson).

**Environmental and Historical Context**

Southwestern North America supported a rich megaherbivore fauna during the Pleistocene. This fauna was largely extinct by the beginning of the Holocene, approximately 11,500 YPB. The hypothesis that most of these species (mammoth, mastodon, desert camel, bison, horse, and others) were hunted to extinction by Paleoindians is supported by remains at Clovis kill sites across the region [79, 80]. Late Pleistocene megaherbivores probably inhabited open grassland, scrub, piñon-juniper, and pine-oak woodland habitats with abundant fine fuel beds of grasses and forbs [81, 82]. The distribution of these vegetation types has contracted since the climate became hotter and drier following the melting of the Laurentide Ice Sheet. Today, these vegetation types are generally found between 1,100 and 2,200 masl; the pattern of distribution of surviving large herbivores is similar.

From the beginning of the Holocene until widespread Euro-American settlement, populations of large herbivores persisted across the region [83-85]. They included pronghorn antelope (*Antilocapra americana*), desert bighorn sheep (*Ovis canadensis nelsoni*), white-tailed deer (*Odocoileus virginianus*), mule deer (*Odocoileus hemionus*), and bison (*Bison bison*). These species were migratory, consuming a range of herbaceous and woody vegetation depending on the species, season, precipitation, and local vegetation complex [82, 86, 87].

Euro-American settlement of the Southwest in the late 1800s brought two further, but dramatic changes to the megaherbivore fauna of the region. First, land-use change and hunting drastically reduced populations of large, native Holocene grazers [85, 88]. Second, settlers introduced large numbers of livestock, especially cattle and sheep. By the 20^th^ century, native megaherbivores had largely been replaced by non-native species throughout the American Southwest.

**Changing relationships of vegetation, herbivory, and fire**

For millennia, wildfire has had large impacts on the abundance and distribution of vegetation and fuels in large-herbivore habitats of southwestern North America [89]. Fire and climate are closely related in the Southwest. Fuel builds up in wet years, and regionally-synchronous fires occur during anomalously dry years [90]. The fire cycle is not well-documented during the cool and wet late-Pleistocene. Limited evidence from charcoal in sediments suggests that fires did occur during this period [91, 92], but fire activity increased dramatically in the early Holocene [92]. This increase was probably due to the hotter and drier climate following glacial melt; it is also possible that loss of megaherbivores led to increased accumulation of fuel, and resulted in more intense or frequent fire [16].

From the early Holocene to the late 1800s the fire regime was characterized by frequent low-intensify surface fires. This changed with widespread introduction of livestock and federally-funded suppression of fire. Euro-American settlement brought many thousands of head of sheep, goats, and cattle to both sides of the US-Mexico borderlands. For example, in the state of Arizona livestock numbers surged from just 37,000 head in 1870 to 1.5 million cattle and 1 million sheep at the peak of the Cattle Boom in the 1890s [93]. Grazing of domestic livestock at high density caused dramatic reduction of fine fuels and was associated with reduced prevalence of low-intensity surface fires across the Southwest, as evidenced in tree-ring fire-scar chronologies [94, 95]. While this pattern of livestock introduction and wildfire cessation began in the U.S. in the 1880s, it did not occur until several decades later in Mexico when the *ejidos* or communal land holdings formed under the 1934 Agrarian Reform Act, and resulted in heavy stocking of domestic livestock in formerly-desolate regions of the country [95, 96]. The result was virtual elimination of fire from the region for over a century. The disruption of the frequent surface fire regime has unintentionally led to a substantial build-up of live and dead fuels.

Recently, the combination of increasing aridity with poor livestock prices and a recent surge in “recreational ranching” have caused the breakup of large working ranches across the region and a drop in numbers of domestic livestock [93, 97, 98]. For example, the number of livestock in the Trans-Pecos region of west Texas declined from 400,000 to 200,000 animal units between 1980 and 2018 ([www.nass.usda.gov](http://www.nass.usda.gov)). There have been similar changes on the other side of the border in northern Mexico, where the breakup of the *ejidos* in 1997 allowed communal Mexican landowners to similarly break up and sell off their lands as well [99].

Regional declines in domestic livestock grazing, coupled with over a century of woody fuel loading under a continually-hotter and drier climate, are leading to a new era of fire—one characterized by much larger, more intense wildfires [100]. As a result, fire risk has become one of the key environmental, economic, and safety concerns in the American Southwest today.

**Rewilding native megaherbivores in southwestern North America**

Public and private land managers are currently involved in large-scale rewilding of native herbivores on both sides of the US-Mexico borderlands. These efforts aim to restore ecosystem function, improve land productivity, and increase income. They could provide the added benefit of mitigating fire risk in areas that are now more vulnerable than ever to anomalous large-scale wildfires. Rewilding in this region provide real-world, working example of native herbivore restorations at several sites across a large region.

Declines in traditional ranching due to both climatic and economic changes have stimulated a shift in private land-use towards wildlife management [101]. Hunting permits for large herbivores such as desert bighorn sheep range from $60,000 to over $300,000 USD for one permit. Permit costs for other large herbivores are similarly high and provide a major economic incentive for private land owners to join public wildlife managers in restoring native herbivores. Texas Parks and Wildlife Department managers have continuing restoration projects for pronghorn antelope, desert bighorn sheep, elk, and whitetail and mule deer on both the Black Gap and Elephant Mountain Wildlife Management areas, as well as on several private ranches [phone interviews with L. A. Harveson, A. Miller, S. Gray]. Likewise, on the Mexican side of the border in Coahuila, the Cemex Corporation has reintroduced all four of these native species to the Sierra del Carmen, and individual ranchers have reintroduced mule and whitetail deer in the nearby Serranías del Burro and Sierra Encantada ranges [phone interview, B. Mckinney]. In all cases, individuals from regionally stable source-brood populations have been used to repopulate areas that once served as native habitat for these species, minimizing effects of genetic outcrossing.

The effects of rewilding on vegetation, fuels, and fire regimes in the region have yet to be assessed. However, there is evidence from herbivore-exclusion experiments that these grazing and browsing ungulates can reduce plant biomass significantly within their current distributions in the Southwest [102, 103]. Moreover, the well-documented decline in fire frequency from domestic livestock grazing suggests that the reintroduction of large, native grazers and browsers has the potential to mitigate fire wildfire risk, especially if implemented in conjunction with fuel hazard reductions of larger woody fuels. Moreover, there are reasons to expect impacts from native herbivores will be more evenly distributed across vegetation than that of livestock. Cattle principally graze on graminoids in the Southwest, with some affinity for shrubby species such as mesquite (*Prosopis spp.*) [104]. Domestic sheep and goats graze more intensively on forbs and woody taxa [105]. In contrast, native herbivores display wider forage preferences. Desert bighorn sheep diets consist in 50% browse, 35% forbs, 11% grasses [106]. Native whitetail and mule deer also browse on a mixture of herbaceous and woody taxa, as do elk and antelope [107, 108]. The more mixed diet of native herbivores than domestic livestock, especially cattle, suggests that rewilding native herbivores could modify not only the herbaceous fuel bed, but also woody shrubs and trees through browsing. This would be especially true at sites such as the Sierra del Carmen in Coahuila, Mexico, where four different species of native herbivores have been reintroduced across one mountain range [phone interview, B. Mckinney].

Grazing pressure in this and in other rewilding locations is largely unknown, and the question remains whether restocking rates of native herbivore will attain levels at which they significantly alter fine and other fuel loadings to mitigate fire risk. Nor do we yet understand the extent to which existing native top predators including mountain lions (*Puma concolor*) and American black bear (*Ursus americanus*) will influence herbivore abundance or modify their effects on the vegetation and fuel complex. Overgrazing is also a concern, as evidenced by the widespread overgrazing by invasive non-native Barbary sheep (Aoudad) throughout west Texas rangelands. Nevertheless, the native megaherbivore rewilding efforts across the American Southwest, in a region where animals roam freely across an international border, provide an exciting opportunity to assess the effectiveness of rewilding, restoration of ecosystem function, sustainable development, and reduction of the threat of wildfire in a fire-adapted landscape.

**E. Case study 2: Asian Water Buffalo (*Bubalus bubalis*) in northern Australia**

**Summary**

- The Asian water buffalo (*Bubalus bubalis*) is a novel megaherbivore in the northern Australia savanna biome, filling a mixed-feeder niche that has been vacant since the Pleistocene megafaunal extinctions.
- Buffalo increased to high densities in Kakadu National Park between the 1950s and 1980, after which their numbers plummeted during a targeted eradication program.
- High densities of buffaloes affect fire regimes and plant and animal communities, as well as floodplain hydrology.
- Buffalo removed most of the surface vegetation from freshwater floodplains. This was associated with reduced fire activity. Subsequent removal of buffalo from these areas was accompanied by more frequent, larger fires.
- Historical reconstruction suggests that the effects of buffalo were smaller in eucalypt savanna, but that they did reduce fire activity by consuming grassy fuels. They also browsed and trampled juvenile trees. Removal of buffalo from heavily-impacted areas appears to have initiated a grass-fire cycle, suppressing recruitment of juvenile trees and further promoting annual grasses.
- Experiments manipulating buffalo show that there were fewer savanna trees in an area where buffalo were removed than where they remained, due to altered competitive relationships between trees and grasses, as well as effects on fuel loads and resulting fire regimes.
- Synthesis of available data suggest that high densities of buffalo are associated with reduced fire activity, but that this comes at a heavy environmental cost. Removal of buffalo did not restore savanna vegetation to its original condition.
- Attitudes to the trade-offs of ecological costs and benefits are culturally framed.
- Implications for rewilding are that buffalo are not perfect analogues for extinct marsupial megafauna, because they have hooves and their trampling and wallowing has substantial eco-hydrological impacts.

**
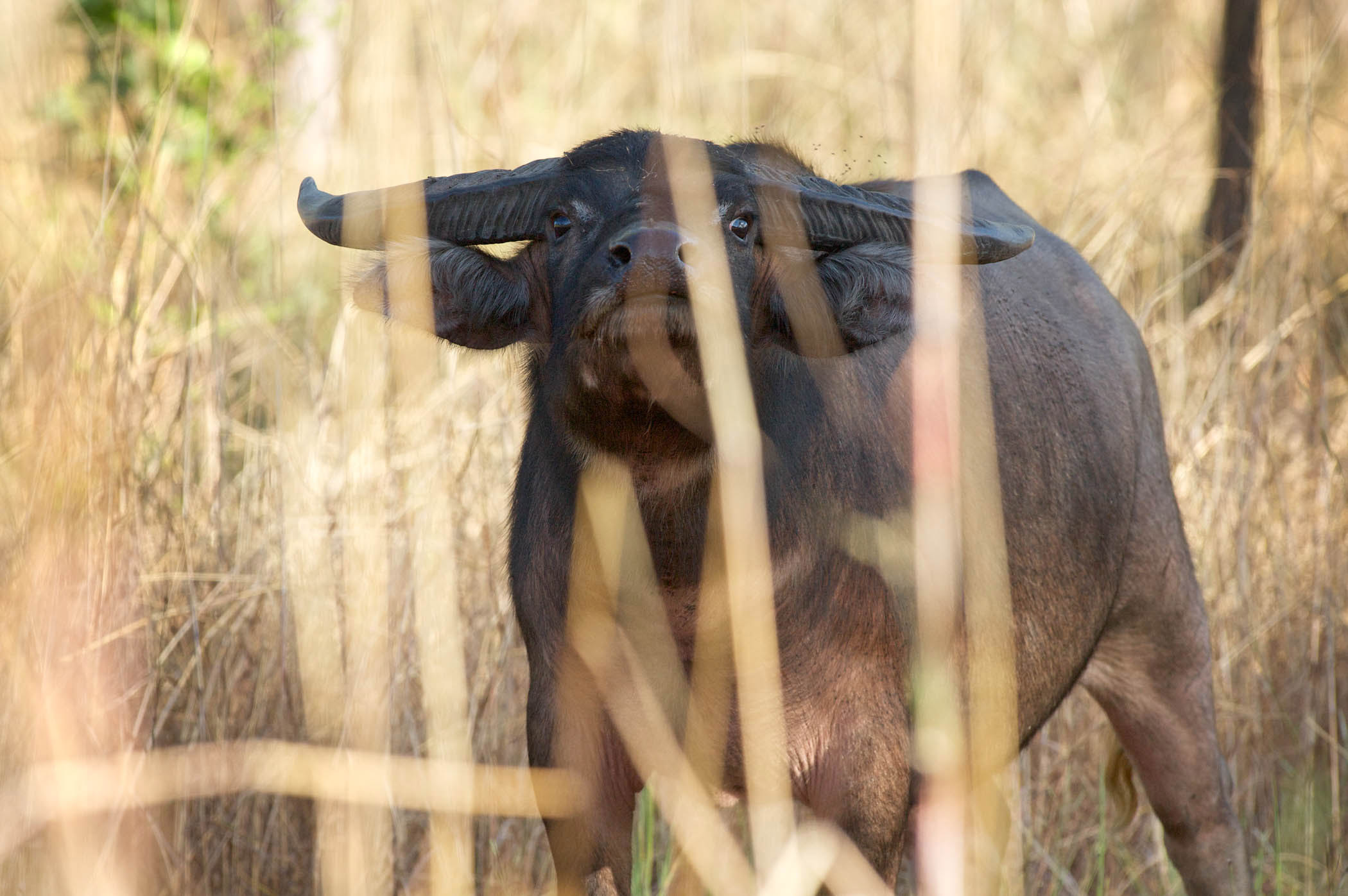
**

Figure S2. Asian water buffalo *Bubalus bubalis* in northern Australia (photo credit: David Hancock]

**Environmental and historical context**

The Asian water buffalo (*Bubalus bubalis*) was one of many species of ungulates introduced to Australia by Europeans. It is around 30 times larger than the largest living marsupial mixed feeders (kangaroos), indicating it may occupy a niche made vacant by the Pleistocene megafauna extinctions [109]. Buffalo were introduced to monsoonal north Australia in the 1820s and became feral. Initially densities were low but increased, especially from 1960 to a peak in 1980 [110, 111]. Numbers then plummeted as a result of the Brucellosis and Tuberculosis Eradication Campaign (BTEC), implemented between 1980 and 1994 to allow export of Australian beef. Buffalo were largely eliminated from Kakadu National Park and the surrounding region. A few herds remained further east in Arnhem Land, and are gradually re-entering the Kakadu region [111].

**Effects on vegetation of fire regimes, herbivory and their interactions**

The greatest impact of buffalo was on the floodplains of the Alligator Rivers Region [111], where they modified the hydrology and removed most of the surface vegetation. Buffalo consume up to 30 kg dry matter per day and are mixed feeders, switching from a diet of primarily grass in the growing season to browse in the dry season [109].

Buffalo reduce grassy fuels in savanna woodlands [48, 112], and they also create tracks that break the continuity of grassy fuels [112]. Consistent with these reductions in grass-fuel biomass and continuity, buffalo presence has been correlated with lower fire activity, especially on the floodplain [113, 114]. The effects of buffalo on woody vegetation in savanna woodlands were more difficult to detect than their effects on floodplain vegetation [111, 114, 115]. This was possibly because while buffalo can directly damage woody vegetation through trampling and browsing, they can also enhance woody plant recruitment by decreasing competition from grasses and reducing the frequency and intensity of fire. The net effect on woody vegetation may be small [112, 114].

**Other environmental and social impacts**

Buffalo created channels and damaged natural levees, allowing salt water to inundate and kill salt-sensitive *Melaleuca* forests [111, 116, 117]. Removing buffalo from the region caused further changes to the vegetation. Petty et al. (2007) [111] state that the floodplain vegetation largely reverted to its original state, but Bowman et al. (2010) [117] detected a legacy effect on salinised areas.

The introduction and subsequent eradication of buffalo initiated a series of demographic effects in savanna tree populations through changing ground-level biomass, which altered competitive relationships as well as fuel loads and resulting fire regimes [27, 111, 118]. For example, there were fewer juvenile trees in an area where buffalo were removed than a comparable area where they remained [27]. The altered fire regimes, to which buffalo introduction and eradication probably contributed, were also associated with severe declines in small native mammals across much of northern Australia, including Kakadu National Park [119, 120] (Woinarski et al. 2010; Lawes et al. 2015).

Buffalo represent a significant reservoir and vector of livestock diseases such as brucellosis and tuberculosis. They can also be a threat to public safety because they can attack people and cause serious collisions with motor vehicles (Bowman and Corbett in press).

**Social Consequences**

The wetlands of the Kakadu region are important economically and culturally. The adverse effects of buffalo on the floodplains were of concern to both the European and Indigenous communities [111, 117]. However, Indigenous people also value the buffalo for subsistence hunting and have incorporated it into their culture. Because of this, they disputed the goal of eradication [121].

**Management responses**

The environmental benefits of removing large populations of buffalo from the Kakadu area were essentially a by-product of the BTEC campaign. However, there were earlier efforts to reduce buffalo numbers for environmental reasons, by managers of a conservation reserve that was later incorporated into Kakadu National Park [111].

Managers of Kakadu National Park employ extensive dry season burning with the aim of reducing fuel loads and the incidence of late dry season fires [122]. However, this approach is expensive to implement and has not been effective in meeting performance thresholds relating to limiting effects of severe fires on vegetation and maintaining significant areas of long unburnt habitat [111]. Land managers are faced with a complex balancing act involving differing, and sometime conflicting, cross-cultural views about the role, value and appropriate densities of buffalo in northern Australia.

**Implications for re-wilding**

Buffalo are not perfect analogues for the extinct megafauna: their hard hooves, wallowing and track-formation behavior create effects which are unlike those of living or (presumably) extinct marsupial large-herbivores, and have particularly large impacts on floodplain vegetation and hydrology, especially when buffalo are present at high densities. Buffalo do, however, provide some benefits for Indigenous people and they can potentially reduce fire activity. Attitudes to buffalo, and tolerance of their adverse effects, differs according to cultural perspective.

**Fig.** Timeline showing major changes in fire and megafauna in the Kakadu region. The black segment in the buffalo timeline indicates increasing buffalo densities to a peak around 1980.

**F. Case study 3: White rhino in Southern Africa.**

**
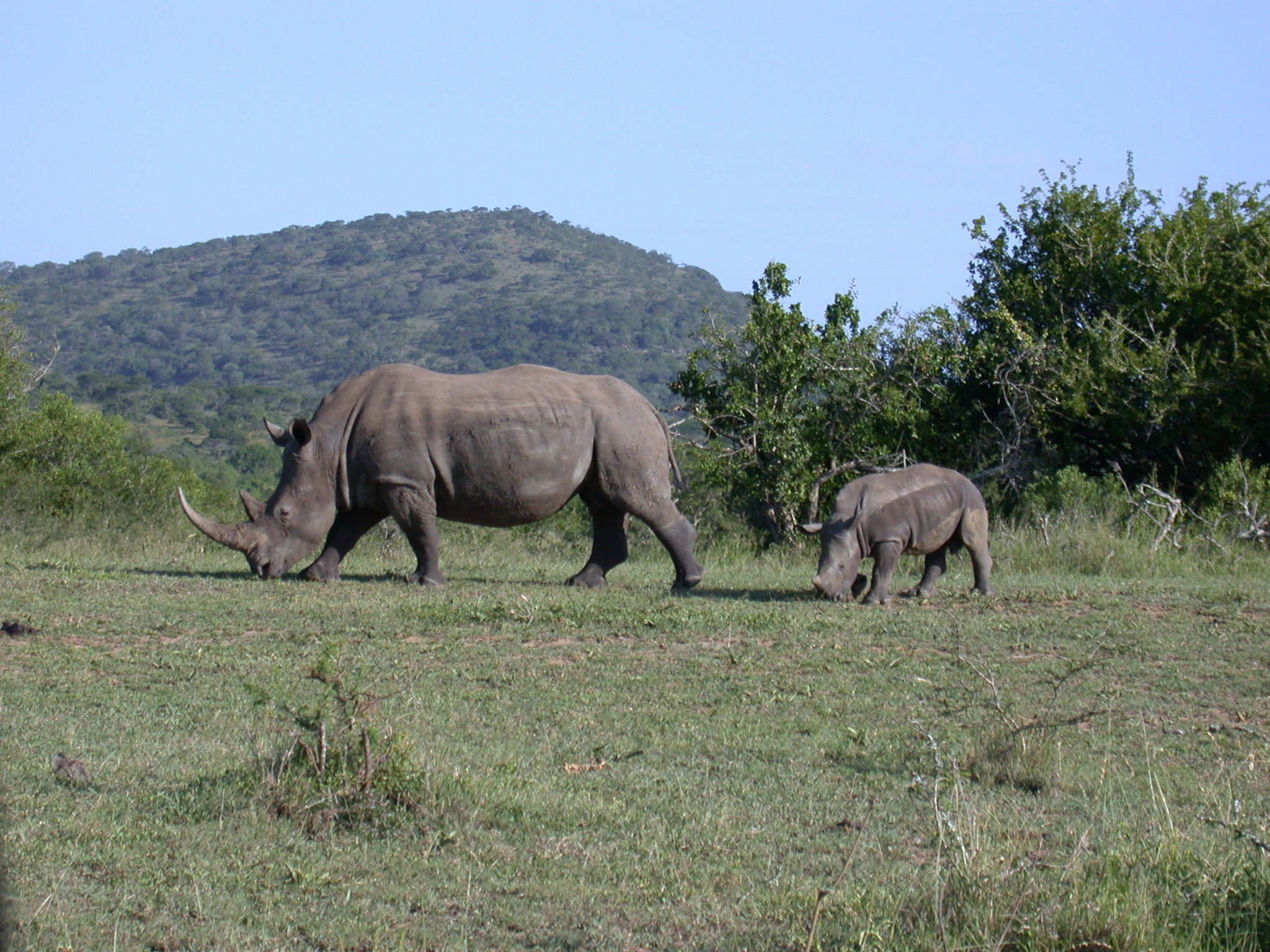
**

**Large-scale patterns of herbivory and fire in African savannas.**

In African savannas there is a functional distinction between more fertile savannas, which tend to be found in arid environments, and dystrophic savannas, which are often found at higher rainfall [123]. Although fire and herbivory are present across the region the dominant consumer of plant biomass is herbivory in arid/eutrophic environments, and fire in mesic/dystrophic environments [124]. The switch from herbivore to fire dominance is locally affected by soil texture, in general herbivores are most important below ~500mm mean annual rainfall (MAR) and fire dominates above ~800mm MAR. Between 500 and 800mm MAR either fire or herbivory may be the dominant control on vegetation biomass [125]. Many of Africa’s most important conservation areas and national parks are within this intermediate high-fire/high-herbivory region, which covers a large area. Notably, it is rare to find sites with intermediate consumption by both herbivores and fire. That is, similar ecosystems contain either many herbivores, or are extensively burned [125]. This supports the idea that the dominant consumer in many African ecosystems can change in response to factors such as drought, disease, or management [126]. It also suggests that herbivory can control fire regimes and reduce fire extent through a large area of the African savannas.

**Evidence that variations in herbivore numbers affected fire regimes: a century of change in Hluhluwe iMfolozi Park**

Hluhluwe iMfolozi park is a small fenced reserve in Kwa-Zulu Natal, South Africa, where MAR varies from ~550 to ~900mm. Soil fertility is generally high, so the park is in the environmental range that allows either fire or herbivory to be the major consumer. It has a well-documented history attesting to large variations in grazer numbers over the more than 100 years since its proclamation [127]. Moreover, scientific research into fire and herbivory have provided insights into how these two above-ground consumers interact.

Spatially comparisons provide evidence for suppression of fire by grazing animals. Dung counts are negatively correlated with the frequency of fire [128], indicating that herbivores/grazing animals could reduce fire spread. Moreover, frequent fire occurs only where the representation of short grazing lawns drops below 10% [54]. This indicates a strong effect of fuel connectivity – which is broken by grazing lawns – on fire spread.

The effect of herbivores on fire regimes is also supported by monitoring of the ecological consequences of changes in herbivore numbers. When the iMfolozi portion of the park was first proclaimed in 1897 it supported a near-complete complement of large mammals, except that hunting during the 19^th^ century had reduced the numbers of buffalo, and extirpated white rhino and elephant [127]. Herbivore biomass was decimated in the early 1930s as a measure to control tsetse fly. Aerial photographs show that a substantial area burned each year [Berry and Macdonald *unpubl. report*], despite active fire suppression, indicating that there might have been a positive response of fire to culling of large herbivores. After tsetse fly was eradicated herbivore biomass recovered quickly through recolonization, and peaked in the early 1970’s atapproximately 8,200kg/km^2^, including 1550 white rhino [129]. At this time fire in the park was highly restricted, never burning more than 20% of the total area [130].

Culling recommenced in the late 1960’s and early 1970s due to concerns that herbivores were over-abundant and causing low grass basal cover that exacerbated erosion. Culling concentrated on the five major grazing ungulates: white rhino, wildebeest, zebra, buffalo and impala [131]. By 1981 large-herbivore biomass in the park was halved to ~4,000kg/km^2^, with white rhino numbers around 600. Although there was a severe drought in the early 1980’s mortality was minor. When the drought broke in 1985 the park experienced the most extensive burns ever recorded: >50% of the park burned for 5 consecutive years [130].

However, culling also slowed after the drought, and herbivore numbers recovered swiftly, especially among the large ungulates and megaherbivores [127]. White rhino numbers are now over 1500 in iMfolozi, and over 3000 in the whole park, and elephant (reintroduced in the 1980s) and buffalo numbers have also increased. Since the 1990s the area burned has ranged from 10 to ~40%, generally increasing over the last decade, and has never reached the highs of over 50% burned in the 1980’s when animal numbers were low [130].

Probably, the park has switched from a period of low herbivores/high fire in the 1930s, to a period of high herbivores/low fire in the 1970s, back to low herbivores/high fire in the 1980s. It is highly likely that the current high abundance of rhino, buffalo, and elephant are holding the area burned at low levels (albeit higher than experienced during the peak herbivory of the 1970s). Variations in rainfall have also been important determinants of fire in this system. During years of high rainfall more herbivores are required to control low fuel loads, and during droughts even low herbivore numbers could be sufficient to switch fire off [132]. Once rainfall variability has been accounted for, however, herbivory still affects area burned [130].

The consequences for vegetation composition of this switch from fire-dominance to herbivore-dominance have been beautifully shown by Bond and Smythe [126]. They noted that in Hluhluwe iMfolozi park there was turnover in tree species composition at different demographic stages: the dominant canopy trees were herbivore-adapted *Acacia nilotica* species, while the dominant juvenile trees were fire-adapted *Acacia karroo*. They proposed that the *Acacia nilotica* canopy trees established in the 1960’s when there was far less fire and higher densities of short-grass specialist grazers. Analysis of tree rings has since confirmed that *A. nilotica* established largely in the late 1960’s to early 1970’s when rhino densities peaked and fire was at its lowest recorded levels, with no recruitment of *A. nilotica* under the current environmental conditions; most of the *A. karroo* trees established in the early 1990’s, and recruitment is continuing under the current fire regime [128]. The consequences of these switches from fire-prone, to herbivore-conditioned ecosystems for other aspects of biodiversity are still to be explored, but clearly, if management aims to use herbivores to control fire regimes, then cascading effects on ecosystems should also be considered.

**Experimental evidence for the role of different herbivores in controlling fire regimes**

The data summarised above are largely correlative, and it is difficult to use them to determine which herbivore species have the strongest effects on fire in the park. Clearly, grazing animals are likely to have bigger impacts than browsers, but are impala as effective as white rhino in altering fuel connectivity and controlling fire spread? Moreover, the large range of rainfall experienced by the park allows us to determine whether efforts to control fire through trophic rewilding might be constrained by environmental conditions.

Herbivores have no impact on fire weather or sources of ignition, so their control of fire operates through their effects on fuel loads, or more importantly, fuel connectivity. It follows that changes in fire size due to herbivory would be the mechanism by which herbivores affect fire regimes in grassy ecosystems. Large herbivores like elephant and buffalo consume tall grass and reduce fuel loads, but short-grass specialists, like white rhino or wildebeest, may have even more influence on fir, through their ability to create and maintain grazing lawns: continuously grazed short-grass patches too short to carry fire.

A landscape-scale experiment tested the role of white rhino relative to other herbivores in altering fuel connectivity and affecting patterns of fire [133]. Rhinos are removed from the park for re-introduction to other conservation areas. Waldram *et al.* located several recent removal areas in the south (arid ~600mm MAR) and north (mesic ~800mm MAR) of the park and compared these with non-removal areas. They measured grass height around extant rhino wallows, and wallows without rhinos (although other animals might still use these habitats), and they used Landsat TM data to quantify the size and patchiness of the fire scars in the area around the wallows.


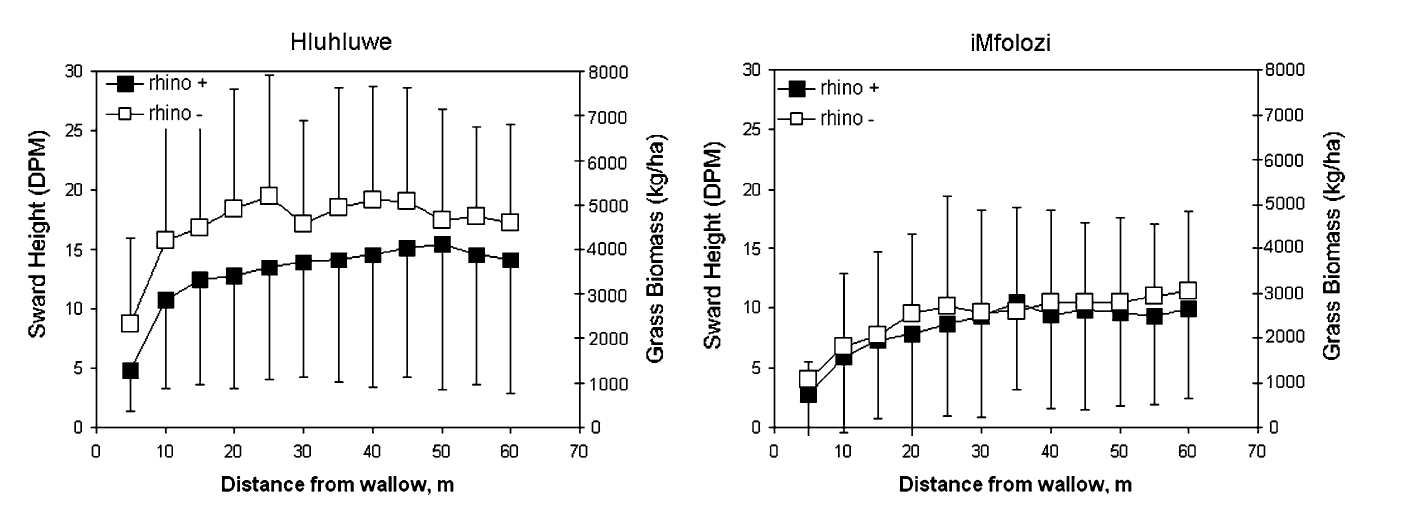


*From Waldram et al (2008): changes in sward height around wallows with and without rhinos at high (Hluhluwe) and low (iMfolozi) rainfall sites. At low rainfall other herbivores can maintain short grass in the absence of rhino, but at high rainfall rhino are required to maintain short-grass patches.*

These comparisons showed that rhinos significantly increase the proportion of short grass in a landscape. At low-rainfall (~600mm MAR) other animals were able to maintain short-grass habitat in the absence of rhinos, but at high rainfall (~800mm MAR) tall grasses dominated when rhinos were removed. Under these conditions, other grazers disappeared.

Fires were always significantly larger when rhinos were removed, but at high rainfall fires increased 50-fold, from approximately 10ha to 500ha on average. In contrast, fires in the low-ranfall iMfolozi were ~4 times larger when rhinos were removed. These results demonstrate that large, “keystone” megaherbivores have disproportionate effects on ecosystem properties, including fire.


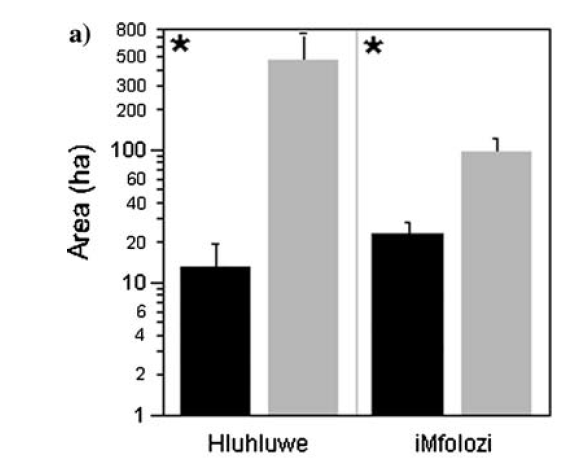


*Figure: Impacts of rhino removals on fire size at high rainfall (Hluhluwe) and low rainfall (iMfolozi) locations. Black represents mean area of each fire with rhinos, and grey the area when rhinos were removed.*

Given that rhino were once common across southern and eastern Africa [134], their role in maintaining short-grass ecosystems and reducing fire size might have been important at regional scales. Evaluation of rhino re-introductions into Kruger National Park showed that across high- and low-nutrient geologies, rhino increased the area of short-grass by a factor of close to 2, and the number of lawns by a factor of 20; short grass areas increased from 15% to 20% on granites and from 6 to 11 % on basalts [135]. Whether these reductions in fuel continuity are sufficient to reduce fire spread has not yet been tested, but given the finding that when short lawn-grass ecosystems increase above 10% fire rapidly drops out of the ecosystem [132], this seems likely.

**Implications for rewilding**

- Effectiveness of herbivore impact on fire depends on the rainfall/soils, as well as the type of herbivore
- Rewilding for fire management will need to consider other ecosystem consequences (eg in the 1960s’ when fire was at its lowest, there were concerns about erosion risk, certain animals/plants being excluded, etc).

**References**

[1] Baker, A. G., Bhagwat, S. A. & Willis, K. J. 2013 Do dung fungal spores make a good proxy for past distribution of large herbivores? *Quaternary Science Reviews* **62**, 21-31. (DOI:<http://dx.doi.org/10.1016/j.quascirev.2012.11.018>).

[2] Johnson, C. N., Rule, S., Haberle, S. G., Turney, C. S. M., Kershaw, A. P. & Brook, B. W. 2015 Using dung fungi to interpret decline and extinction of megaherbivores: problems and solutions. *Quaternary Science Reviews* **110**, 107-113. (DOI:<http://dx.doi.org/10.1016/j.quascirev.2014.12.011>).

[3] Gill, J. L., McLauchlan, K. K., Skibbe, A. M., Goring, S., Zirbel, C. R. & Williams, J. W. 2013 Linking abundances of the dung fungus Sporormiella to the density of bison: implications for assessing grazing by megaherbivores in paleorecords. *Journal of Ecology* **1125-1136**.

[4] Raczka, M. F., Bush, M. B., Folcik, A. M. & McMichael, C. H. 2016 Sporormiella as a tool for detecting the presence of large herbivores in the Neotropics. *Biota Neotropica* **16**, e20150090. (DOI:doi.org/10.1590/1676-0611-BN-2015-0090).

[5] Wood, J. R., Wilmshurst, J. M., Worthy, T. H. & Cooper, A. 2011 Sporormiella as a proxy for non-mammalian herbivores in island ecosystems. *Quaternary Science Reviews* **30**, 915-920. (DOI:<http://dx.doi.org/10.1016/j.quascirev.2011.01.007>).

[6] Wood, J. R., Wilmshurst, J. M., Turney, C. S. M. & Fogwill, C. J. 2016 Palaeoecological signatures of vegetation change induced by herbivory regime shifts on subantarctic Enderby Island. *Quaternary Science Reviews* **134**, 51-58. (DOI:<http://dx.doi.org/10.1016/j.quascirev.2015.12.018>).

[7] Graham, R. W., Belmecheri, S., Choy, K., Culleton, B. J., Davies, L. J., Froese, D., Heintzman, P. D., Hritz, C., Kapp, J. D., Newsom, L. A., et al. 2016 Timing and causes of mid-Holocene mammoth extinction on St. Paul Island, Alaska. *Proceedings of the National Academy of Sciences* **113**, 9310-9314.

[8] Raczka, M. F., Bush, M. B. & De Oliveira, P. E. 2017 The collapse of megafaunal populations in southeastern Brazil. *Quaternary Research*, 1-16. (DOI:10.1017/qua.2017.60).

[9] Perry, G. L. W., Wilmshurst, J. M., McGlone, M. S., McWethy, D. B. & Whitlock, C. 2012 Explaining fire-driven landscape transformation during the Initial Burning Period of New Zealand's prehistory. *Global Change Biology* **18**, 1609-1621. (DOI:10.1111/j.1365-2486.2011.02631.x).

[10] Burney, D. A., Robinson, G. S. & Burney, L. P. 2003 *Sporormiella* and the late Holocene extinctions in Madagascar. *Proceedings of the National Academy of Science of the USA* **100**, 10800-10805.

[11] van der Kaars, S., Miller, G. H., Turney, C. S. M., Cook, E. J., Nürnberg, D., Schönfeld, J., Kershaw, A. P. & Lehman, S. J. 2017 Humans rather than climate the primary cause of Pleistocene megafaunal extinction in Australia. *Nature Communications* **8**, 14142. (DOI:10.1038/ncomms14142

<http://www.nature.com/articles/ncomms14142#supplementary-information>).

[12] Rule, S., Brook, B. W., Haberle, S. G., Turney, C. S. M., Kershaw, A. P. & Johnson, C. N. 2012 The aftermath of megafaunal extinction: Ecosystem transformation in Pleistocene Australia. *Science* **335**, 1483-1486. (DOI:10.1126/science.1214261).

[13] Johnson, C. N., Rule, S., Haberle, S. G., Kershaw, A. P., McKenzie, G. M. & Brook, B. W. 2016 Geographic variation in the ecological effects of extinction of Australia's Pleistocene megafauna. *Ecography* **39**, 109-116. (DOI:10.1111/ecog.01612).

[14] Rozas-Davila, A., Valencia, B. G. & Bush, M. B. 2016 The functional extinction of Andean megafauna. *Ecology* **97**, 2533-2539. (DOI:10.1002/ecy.1531).

[15] Gill, J. L., Williams, J. W., Jackson, S. T., Donnelly, J. P. & Schellinger, G. C. 2012 Climatic and megaherbivory controls on late-glacial vegetation dynamics: a new, high-resolution, multi-proxy record from Silver Lake, Ohio. *Quaternary Science Reviews* **34**, 66-80. (DOI:<http://dx.doi.org/10.1016/j.quascirev.2011.12.008>).

[16] Gill, J. L., Williams, J. W., Jackson, S. T., Lininger, K. B. & Robinson, G. S. 2009 Pleistocene megafaunal collapse, novel plant communities, and enhanced fire regimes in North America. *Science* **326**, 1100-1103. (DOI:10.1126/science.1179504).

[17] Jeffers, E. S., Bonsall, M. B., Watson, J. E. & Willis, K. J. 2012 Climate change impacts on ecosystem functioning: evidence from an Empetrum heathland. *New Phytologist* **193**, 150-164. (DOI:10.1111/j.1469-8137.2011.03907.x).

[18] Robinson, G. S., Burney, L. P. & Burney, D. A. 2005 Landscape paleoecology and megafaunal extinction in southeastern New York state. *Ecological Monographs* **75**, 295-315.

[19] Perrotti, A. G. 2018 Pollen and Sporormiella evidence for terminal Pleistocene vegetation change and megafaunal extinction at Page-Ladson, Florida. *Quaternary International* **466**, 256-268. (DOI:<https://doi.org/10.1016/j.quaint.2017.10.015>).

[20] hansen, B. C. 2006 Setting the stage: fossil pollen, stomata, and charcoal. In *First Floridians and Last Mastodons: The Page-Ladson site in the Aucilla River* (eds. J. S. Dunbar & S. D. Webb), pp. 159-179. New York, Springer.

[21] Jeffers, E. S., Bonsall, M. B. & Willis, K. J. 2011 Stability in Ecosystem Functioning across a Climatic Threshold and Contrasting Forest Regimes. *PLoS ONE* **6**, e16134. (DOI:10.1371/journal.pone.0016134).

[22] Burney, D. A. 1993 Late Holocene Environmental Changes in Arid Southwestern Madagascar. *Quaternary Research* **40**, 98-106.

[23] Froyd, C. A., Coffey, E. E. D., van der Knaap, W. O., van Leeuwen, J. F. N., Tye, A. & Willis, K. J. 2014 The ecological consequences of megafaunal loss: giant tortoises and wetland biodiversity. *Ecology Letters* **17**, 144-154. (DOI:10.1111/ele.12203).

[24] Eldridge, D. J., Poore, A. G. B., Ruiz-Colmenero, M., Letnic, M. & Soliveres, S. 2016 Ecosystem structure, function, and composition in rangelands are negatively affected by livestock grazing. *Ecological Applications* **26**, 1273-1283. (DOI:10.1890/15-1234).

[25] Davies, K. W., Bates, J. D., Svejcar, T. J. & Boyd, C. S. 2010 Effects of long-term livestock grazing on fuel characteristics in rangelands: an example from the sagebrush steppe. *Rangeland Ecology & Management* **63**, 662-669. (DOI:10.2111/rem-d-10-00006.1).

[26] Singer, F. J. & Harter, M. K. 1996 Comparative effects of elk herbivory and 1988 fires on northern Yellowstone National Park grasslands. *Ecological Applications* **6**, 185-199. (DOI:10.2307/2269563).

[27] Werner, P. A., Cowie, I. D. & Cusack, J. S. 2006 Impact of feral water buffalo on juvenile trees in savannas of northern Australia: An experimental field study in Kakadu National Park. *Australian Journal of Botany* **54**, 283-296.

[28] Van Auken, O. W. 2000 Shrub invasions of North American semiarid grasslands. *Annual Review of Ecology and Systematics* **31**, 197-215. (DOI:10.1146/annurev.ecolsys.31.1.197).

[29] Burkepile, D. E., Fynn, R. W. S., Thompson, D. I., Lemoine, N. P., Koerner, S. E., Eby, S., Hagenah, N., Wilcox, K. R., Collins, S. L., Kirkman, K. P., et al. 2017 Herbivore size matters for productivity-richness relationships in African savannas. *Journal of Ecology* **105**, 674-686. (DOI:10.1111/1365-2745.12714).

[30] Kimuyu, D. M., Sensenig, R. L., Riginos, C., Veblen, K. E. & Young, T. P. 2014 Native and domestic browsers and grazers reduce fuels, fire temperatures, and acacia ant mortality in an African savanna. *Ecological Applications* **24**, 741-749. (DOI:10.1890/13-1135.1).

[31] Elmore, R. D., Bielski, C. H., Fuhlendorf, S. D., Hovick, T. J., Starns, H. D., Thacker, E. T. & Twidwell, D. L. 2017 *Managing fuels while enhancing prairie-chicken habitat. Final Report*. Stillwater, OK, Oklahoma State University.

[32] Staver, A. C. & Bond, W. J. 2014 Is there a 'browse trap'? Dynamics of herbivore impacts on trees and grasses in an African savanna. *Journal of Ecology* **102**, 595-602. (DOI:10.1111/1365-2745.12230).

[33] Kirkpatrick, J. B., Marsden-Smedley, J. B. & Leonard, S. W. J. 2011 Influence of grazing and vegetation type on post-fire flammability. *Journal of Applied Ecology* **48**, 642-649. (DOI:10.1111/j.1365-2664.2011.01962.x).

[34] Pachzelt, A., Forrest, M., Rammig, A., Higgins, S. I. & Hickler, T. 2015 Potential impact of large ungulate grazers on African vegetation, carbon storage and fire regimes. *Global Ecology and Biogeography* **24**, 991-1002. (DOI:10.1111/geb.12313).

[35] Collins, S. L. 1987 Interaction of disturbances in tallgrass prairie - a field experiment. *Ecology* **68**, 1243-1250. (DOI:10.2307/1939208).

[36] Staver, A. C., Bond, W. J., Stock, W. D., van Rensburg, S. J. & Waldram, M. S. 2009 Browsing and fire interact to suppress tree density in an African savanna. *Ecological Applications* **19**, 1909-1919. (DOI:10.1890/08-1907.1).

[37] Coverdale, T. C., Kartzinel, T. R., Grabowski, K. L., Shriver, R. K., Hassan, A. A., Goheen, J. R., Palmer, T. M. & Pringle, R. M. 2016 Elephants in the understory: opposing direct and indirect effects of consumption and ecosystem engineering by megaherbivores. *Ecology* **97**, 3219-3230. (DOI:10.1002/ecy.1557).

[38] Riggs, R. A., Tiedemann, A. R., Cook, J. G., Ballard, T. M., Edgerton, P. J., Vavra, M., Krueger, W. C., Hall, F. C., Bryant, L. D., Irwin, L. L., et al. 2000 Modification of mixed-conifer forests by ruminant herbivores in the blue mountains ecological province. *USDA For. Serv. Pac. Northwest Res. Stn. Res. Pap. PNW-RP*, 1-+.

[39] Johansson, M. U. & Granström, A. 2014 Fuel, fire and cattle in African highlands: traditional management maintains a mosaic heathland landscape. *Journal of Applied Ecology* **51**, 1396-1405. (DOI:10.1111/1365-2664.12291).

[40] Augustine, D. J. & Mcnaughton, S. J. 2004 Regulation of shrub dynamics by native browsing ungulates on East African rangeland. *Journal of Applied Ecology* **41**, 45-58.

[41] Trollope, W. S. W. 1974 Role of fire in preventing bush encroachment in the Eastern Cape *Proceedings of the Annual Congresses of the Grassland Society of Southern Africa* **9**, 67-72. (DOI:10.1080/00725560.1974.9648722).

[42] Daskin, J. H., Stalmans, M. & Pringle, R. M. 2016 Ecological legacies of civil war: 35-year increase in savanna tree cover following wholesale large-mammal declines. *Journal of Ecology* **104**, 79-89. (DOI:10.1111/1365-2745.12483).

[43] Sankaran, M., Augustine, D. J. & Ratnam, J. 2013 Native ungulates of diverse body sizes collectively regulate long-term woody plant demography and structure of a semi-arid savanna. *Journal of Ecology* **101**, 1389-1399. (DOI:10.1111/1365-2745.12147).

[44] Pellegrini, A. F. A., Pringle, R. M., Govender, N. & Hedin, L. O. 2017 Woody plant biomass and carbon exchange depend on elephant-fire interactions across a productivity gradient in African savanna. *Journal of Ecology* **105**, 111-121. (DOI:10.1111/1365-2745.12668).

[45] Churski, M., Bubnicki, J. W., Jedrzejewska, B., Kuijper, D. P. J. & Cromsigt, J. 2017 Brown world forests: increased ungulate browsing keeps temperate trees in recruitment bottlenecks in resource hotspots. *New Phytologist* **214**, 158-168. (DOI:10.1111/nph.14345).

[46] Giljohann, K. M., McCarthy, M. A., Keith, D. A., Kelly, L. T., Tozer, M. G. & Regan, T. J. 2017 Interactions between rainfall, fire and herbivory drive resprouter vital rates in a semi-arid ecosystem. *Journal of Ecology* **105**, 1562-1570. (DOI:10.1111/1365-2745.12768).

[47] Gordijn, P. J., Rice, E. & Ward, D. 2012 The effects of fire on woody plant encroachment are exacerbated by succession of trees of decreased palatability. *Perspect. Plant Ecol. Evol. Syst.* **14**, 411-422. (DOI:10.1016/j.ppees.2012.09.005).

[48] Werner, P. A. 2005 Impact of feral water buffalo and fire on growth and survival of mature savanna trees: An experimental field study in Kakadu National Park, northern Australia. *Austral Ecology* **30**, 625-647.

[49] Booth, C. A., King, G. W. & Sanchez-Bayo, F. 1996 Establishment of woody weeds in western New South Wales. 1. Seedling emergence and phenology. *The Rangeland Journal* **18**, 58-79. (DOI:10.1071/RJ9960058).

[50] Milchunas, D. G. & Lauenroth, W. K. 1993 Quantitative effects of grazing on vegetation and soils over a global range of environments. *Ecological Monographs* **63**, 327-366. (DOI:10.2307/2937150).

[51] Buechner, H. K. & Dawkins, H. C. 1961 Vegetation change induced by elephants and fire in Murchison Falls National Park, Uganda. *Ecology* **42**, 752-766.

[52] Endress, B. A., Wisdom, M. J., Vavra, M., Parks, C. G., Dick, B. L., Naylor, B. J. & Boyd, J. M. 2012 Effects of ungulate herbivory on aspen, cottonwood, and willow development under forest fuels treatment regimes. *Forest Ecology and Management* **276**, 33-40. (DOI:10.1016/j.foreco.2012.03.019).

[53] Madany, M. H. & West, N. E. 1983 Livestock grazing fire regime interactions within montane forests of Zion National Park, Utah. *Ecology* **64**, 661-667. (DOI:10.2307/1937186).

[54] Archibald, S., Bond, W. J., Stock, W. D. & Fairbanks, D. H. K. 2005 Shaping the landscape: Fire-grazer interactions in an African savanna. *Ecological Applications* **15**, 96-109. (DOI:10.1890/03-5210).

[55] McNaughton, S. J. 1984 Grazing lawns - animals in herds, plant form, and coevolution. *American Naturalist* **124**, 863-886. (DOI:10.1086/284321).

[56] Cromsigt, J. & te Beest, M. 2014 Restoration of a megaherbivore: landscape-level impacts of white rhinoceros in Kruger National Park, South Africa. *Journal of Ecology* **102**, 566-575. (DOI:10.1111/1365-2745.12218).

[57] Knapp, A. K., Blair, J. M., Briggs, J. M., Collins, S. L., Hartnett, D. C., Johnson, L. C. & Towne, E. G. 1999 The keystone role of bison in north American tallgrass prairie - Bison increase habitat heterogeneity and alter a broad array of plant, community, and ecosystem processes. *Bioscience* **49**, 39-50.

[58] Fuhlendorf, S. D., Engle, D. M., Kerby, J. & Hamilton, R. 2009 Pyric Herbivory: Rewilding Landscapes through the Recoupling of Fire and Grazing. *Conservation Biology* **23**, 588-598. (DOI:10.1111/j.1523-1739.2008.01139.x).

[59] Scasta, J. D., Duchardt, C., Engle, D. M., Miller, J. R., Debinski, D. M. & Harr, R. N. 2016 Constraints to restoring fire and grazing ecological processes to optimize grassland vegetation structural diversity. *Ecol. Eng.* **95**, 865-875. (DOI:10.1016/j.ecoleng.2016.06.096).

[60] Blackhall, M., Veblen, T. T. & Raffaele, E. 2015 Recent fire and cattle herbivory enhance plant-level fuel flammability in shrublands. *Journal of Vegetation Science* **26**, 123-133. (DOI:10.1111/jvs.12216).

[61] Davies, K. W., Bates, J. D., Boyd, C. S. & Svejcar, T. J. 2016 Prefire grazing by cattle increases postfire resistance to exotic annual grass (*Bromus tectorum*) invasion and dominance for decades. *Ecology and Evolution* **6**, 3356-3366. (DOI:10.1002/ece3.2127).

[62] Hobbs, N. T., Schimel, D. S., Owensby, C. E. & Ojima, D. S. 1991 Fire and grazing in the tallgrass prairie - contingent effects on nitrogen budgets. *Ecology* **72**, 1374-1382. (DOI:10.2307/1941109).

[63] Holdo, R. M., Holt, R. D., Coughenour, M. B. & Ritchie, M. E. 2007 Plant productivity and soil nitrogen as a function of grazing, migration and fire in an African savanna. *Journal of Ecology* **95**, 115-128. (DOI:10.1111/j.1365-2745.2006.01192.x).

[64] Williamson, G. J., Murphy, B. P. & Bowman, D. M. J. S. 2014 Cattle grazing does not reduce fire severity in eucalypt forests and woodlands of the Australian Alps. *Austral Ecology* **39**, 462-468. (DOI:10.1111/aec.12104).

[65] Kerby, J. D., Fuhlendorf, S. D. & Engle, D. M. 2007 Landscape heterogeneity and fire behavior: scale-dependent feedback between fire and grazing processes. *Landsc. Ecol.* **22**, 507-516. (DOI:10.1007/s10980-006-9039-5).

[66] Donaldson, J. E., Archibald, S., Govender, N., Pollard, D., Luhdo, Z. & Parr, C. L. 2018 Ecological engineering through fire-herbivory feedbacks drives the formation of savanna grazing lawns. *Journal of Applied Ecology* **55**, 225-235. (DOI:10.1111/1365-2664.12956).

[67] Waldram, M. S., Bond, W. J. & Stock, W. D. 2008 Ecological engineering by a mega-grazer: White Rhino impacts on a South African savanna. *Ecosystems* **11**, 101-112. (DOI:10.1007/s10021-007-9109-9).

[68] McNaughton, S. J. 1992 The propagation of disturbance in savannas through food webs. *Journal of Vegetation Science* **3**, 301-314. (DOI:10.2307/3235755).

[69] Dubinin, M., Luschekina, A. & Radeloff, V. C. 2011 Climate, Livestock, and Vegetation: What Drives Fire Increase in the Arid Ecosystems of Southern Russia? *Ecosystems* **14**, 547-562. (DOI:10.1007/s10021-011-9427-9).

[70] Trauernicht, C., Murphy, B. P., Portner, T. E. & Bowman, D. M. J. S. 2012 Tree cover–fire interactions promote the persistence of a fire-sensitive conifer in a highly flammable savanna. *Journal of Ecology*, no-no. (DOI:10.1111/j.1365-2745.2012.01970.x).

[71] Smit, I. P. J. & Prins, H. H. T. 2015 Predicting the Effects of Woody Encroachment on Mammal Communities, Grazing Biomass and Fire Frequency in African Savannas. *Plos One* **10**, 16. (DOI:10.1371/journal.pone.0137857).

[72] O'Connor, T. G., Mulqueeny, C. M. & Goodman, P. S. 2011 Determinants of spatial variation in fire return period in a semiarid African savanna. *International Journal of Wildland Fire* **20**, 540-549. (DOI:10.1071/wf08142).

[73] Savage, M. & Swetnam, T. W. 1990 Early 19th Century fire decline following sheep pasturing in a Navajo ponderosa pine forest. *Ecology* **71**, 2374-2378. (DOI:10.2307/1938649).

[74] Van Auken, O. W. 2009 Causes and consequences of woody plant encroachment into western North American grasslands. *Journal of Environmental Management* **90**, 2931-2942. (DOI:10.1016/j.jenvman.2009.04.023).

[75] Van Auken, O. W. 1993 Size distribution patterns and potential population change of some dominant woody species of the Edwards Plateau region of Texas. *Tex. J. Sci.* **45**, 199-210.

[76] Harrington, G. N., Oxley, R. E. & Tongway, D. J. 1979 The effects of European settlement and domestic livestock on the biological system in poplar box (*Eucalyptus populnea*) lands. *Australian Rangeland Journal* **1**, 271-279.

[77] Noble, J. C., Hik, D. S. & Sinclair, A. R. E. 2007 Landscape ecology of the burrowing bettong: fire and marsupial biocontrol of shrubs in semi-arid Australia. *Rangeland Journal* **29**, 107-119. (DOI:10.1071/rj06041).

[78] Leonard, S., Kirkpatrick, J. & Marsden-Smedley, J. 2010 Variation in the effects of vertebrate grazing on fire potential between grassland structural types. *Journal of Applied Ecology* **47**, 876-883.

[79] Haynes, G. 2009 *American megafaunal extinctions at the end of the Pleiostocene*, Springer.

[80] Grayson, D. K. & Meltzer, D. J. 2002 Clovis hunting and large mammal extinction: a critical rreview of the evidence. *J World Prehist* **16**, 313-359.

[81] Van Devender, T. R. & Spaulding, W. G. 1979 Development of Vegetation and Climate in the Southwestern United States. *Science* **204**, 701.

[82] Connin, S. L., Betancourt, J. & Quade, J. 1998 Late Pleistocene C_4_ plant dominance and summer rainfall in the southwestern United States from isotopic study of herbivore teeth. *Quaternary Research* **50**, 179-193.

[83] Bauer, E. A. 1997 *Big game of North America*, Voyageur Press.

[84] Davis, G. P. 1973 Man and wildlife in Arizona the pre-settlement era, 1823-1864, The UNiversity of Arizona.

[85] Valdez, R. & Krausman, P. R. 1999 *Mountain sheep of North America*, University of Arizona Press.

[86] Rivals, F. & Semprebon, G. M. 2006 A comparison of the dietary habits of a large sample of the Pleistocene pronghorn *Stockoceros onusrosagris* from the Papago Springs Cave in Arizona to the modern *Antilocapra americana*. *Journal of Vertebrate Paleontology* **26**, 495-500.

[87] Nunez, E. E., Macfadden, B. J., Mead, J. I. & Baez, A. 2010 Ancient forests and grasslands in the desert: Diet and habitat of Late Pleistocene mammals from Northcentral Sonora, Mexico. *Palaeogeography, Palaeoclimatology, Palaeoecology* **297**, 391-400. (DOI:<https://doi.org/10.1016/j.palaeo.2010.08.021>).

[88] Wallace, M. C. & Krausman, P. R. 1987 Elk, Mule Deer, and Cattle Habitats in Central Arizona. *Journal of Range Management* **40**, 80-83. (DOI:10.2307/3899367).

[89] Swetnam, T. W. & Betancourt, J. L. 1998 Mesoscale Disturbance and Ecological Response to Decadal Climatic Variability in the American Southwest. *Journal of Climate* **11**, 3128-3147. (DOI:10.1175/1520-0442(1998)011<3128:MDAERT>2.0.CO;2).

[90] Swetnam, T. W. & Betancourt, J. L. 1990 Fire-Southern Oscillation Relations in the Southwestern United States. *Science* **249**, 1017.

[91] Anderson, R. S., Jass, R. B., Toney, J. L., Allen, C. D., Cisneros-Dozal, L. M., Hess, M., Heikoop, J. & Fessenden, J. 2008 Development of the mixed conifer forest in northern New Mexico and its relationship to Holocene environmental change. *Quaternary Research* **69**, 263-275. (DOI:<https://doi.org/10.1016/j.yqres.2007.12.002>).

[92] Holmgren, C. A., Betancourt, J. L. & Rylander, K. A. 2006 A 36,000-yr vegetation history from the Peloncillo Mountains, southeastern Arizona, USA. *Palaeogeography, Palaeoclimatology, Palaeoecology* **240**, 405-422. (DOI:<https://doi.org/10.1016/j.palaeo.2006.02.017>).

[93] Sheridan, T. E. 2001 Cows, Condos, and the Contested Commons: The Political Ecology of Ranching on the Arizona-Sonora Borderlands. *Human Organization* **60**, 141-152. (DOI:10.17730/humo.60.2.991hqu9q6ryf5aav).

[94] Sakulich, J. & Taylor, A. H. 2007 Fire regimes and forest structure in a sky island mixed conifer forest, Guadalupe Mountains National Park, Texas, USA. *Forest Ecology and Management* **241**, 62-73. (DOI:<https://doi.org/10.1016/j.foreco.2006.12.029>).

[95] Poulos, H. M., Villanueva Díaz, J., Cerano Paredes, J., Camp, A. E. & Gatewood, R. G. 2013 Human influences on fire regimes and forest structure in the Chihuahuan Desert Borderlands. *Forest Ecology and Management* **298**, 1-11. (DOI:<https://doi.org/10.1016/j.foreco.2013.02.014>).

[96] Fulé, P. Z. & Covington, W. W. 1996 Changing Fire Regimes in Mexican Pine Forests: Ecological and Management Implications. *Journal of Forestry* **94**, 33-38. (DOI:10.1093/jof/94.10.33).

[97] Eakin, H. & Conley, J. 2002 Climate variability and the vulnerability of ranching in southeastern Arizona

a pilot study. *Climate Research* **21**, 271-281.

[98] Curtin, C. G., Sayre, N. F. & Lane, B. D. 2002 Transformations of the Chihuahuan Borderlands: grazing, fragmentation, and biodiversity conservation in desert grasslands. *Environmental Science & Policy* **5**, 55-68. (DOI:<https://doi.org/10.1016/S1462-9011(02)00020-5>).

[99] Jones Gareth, A. & Ward Peter, M. 2002 Privatizing the commons: reforming the ejido and urban development in Mexico. *International Journal of Urban and Regional Research* **22**, 76-93. (DOI:10.1111/1468-2427.00124).

[100] Littell Jeremy, S., McKenzie, D., Peterson David, L. & Westerling Anthony, L. 2009 Climate and wildfire area burned in western U.S. ecoprovinces, 1916–2003. *Ecological Applications* **19**, 1003-1021. (DOI:10.1890/07-1183.1).

[101] Hurley, K., Brewer, C. & Thornton, G. N. 2015 The role of hunters in conservation, restoration, and management of North American wild sheep. *International Journal of Environmental Studies* **72**, 784-796. (DOI:10.1080/00207233.2015.1031567).

[102] Veblen, K. E., Nehring, K. C., McGlone, C. M. & Ritchie, M. E. 2015 Contrasting Effects of Different Mammalian Herbivores on Sagebrush Plant Communities. *PLOS ONE* **10**, e0118016. (DOI:10.1371/journal.pone.0118016).

[103] Wikeem, B. M. & Pitt, M. D. 1991 Grazing Effects and Range Trend Assessment on California Bighorn Sheep Range. *Journal of Range Management* **44**, 466-470. (DOI:10.2307/4002746).

[104] Clary, W. P. & Pearson, H. A. 1969 Cattle Preferences for Forage Species in Northern Arizona. *Journal of Range Management* **22**, 114-116. (DOI:10.2307/3896193).

[105] McMahan, C. A. 1964 Comparative Food Habits of Deer and Three Classes of Livestock. *The Journal of Wildlife Management* **28**, 798-808. (DOI:10.2307/3798797).

[106] Brewer, C. E. & Harveson, L. A. 2007 Diets of bighorn sheep in the Chihuahuan Desert, Texas *The Southwestern Naturalist* **52**, 97-103. (DOI:10.1894/0038-4909(2007)52[97:DOBSIT]2.0.CO;2).

[107] Anthony Robert, G. & Smith Norman, S. 1977 Ecological Relationships between Mule Deer and White‐Tailed Deer in Southeastern Arizona. *Ecological Monographs* **47**, 255-277. (DOI:10.2307/1942517).

[108] Beale, D. M. & Smith, A. D. 1970 Forage Use, Water Consumption, and Productivity of Pronghorn Antelope in Western Utah. *The Journal of Wildlife Management* **34**, 570-582. (DOI:10.2307/3798865).

[109] Bowman, D. M. J. S., Murphy, B. P. & McMahon, C. R. 2010 Using carbon isotope analysis of the diet of two introduced Australian megaherbivores to understand Pleistocene megafaunal extinctions. *Journal of Biogeography* **37**, 499-505. (DOI:10.1111/j.1365-2699.2009.02206.x).

[110] Skeat, A. J., East, T. J. & Corbett, L. K. 1996 Impact of feral water buffalo. In *Landscape and Vegetation Ecology of the Kakadu Region, Northern Australia* (eds. C. M. Finlayson & I. von Oertzen), pp. 155-177. Dordrecht, Kluwer Academic.

[111] Petty, A. M., Werner, P. A., Lehmann, C. E. R., Riley, J. E., Banfai, D. S. & Elliott, L. P. 2007 Savanna responses to feral buffalo in Kakadu National Park, Australia. *Ecological Monographs* **77**, 441-463.

[112] Trauernicht, C., Murphy, B. P., Tangalin, N. & Bowman, D. M. J. S. 2013 Cultural legacies, fire ecology, and environmental change in the Stone Country of Arnhem Land and Kakadu National Park, Australia. *Ecology and Evolution* **3**, 286-297. (DOI:10.1002/ece3.460).

[113] Russell-Smith, J., Ryan, P. G. & Durieu, R. 1997 A LANDSAT MSS-derived fire history of Kakadu National Park, monsoonal northern Australia, 1980-94: seasonal extent, frequency and patchiness. *Journal of Applied Ecology* **34**, 748-766.

[114] Lehmann, C. E. R., Prior, L. D., Williams, R. J. & Bowman, D. M. J. S. 2008 Spatio-temporal trends in tree cover of a tropical mesic savanna are driven by landscape disturbance. *Journal of Applied Ecology* **45**, 1304-1311.

[115] Bowman, D. M. J. S., Riley, J. E., Boggs, G. S., Lehmann, C. E. R. & Prior, L. D. 2008 Do feral buffalo (*Bubalus bubalis*) explain the increase of woody cover in savannas of Kakadu National Park, Australia? *Journal of Biogeography* **35**, 1976-1988. (DOI:10.1111/j.1365-2699.2008.01934.x).

[116] Finlayson, C. M., Storrs, M. J. & Lindner, G. 1997 Degradation and rehabilitation of wetlands in the Alligator Rivers Region of northern Australia. *Wetlands Ecology and Management* **5**, 19-36.

[117] Bowman, D. M. J. S., Prior, L. D. & De Little, S. C. 2010 Retreating *Melaleuca* swamp forests in Kakadu National Park: Evidence of synergistic effects of climate change and past feral buffalo impacts. *Austral Ecology* **35**, 898-905. (DOI:10.1111/j.1442-9993.2009.02096.x).

[118] Prior, L. D., Brook, B. W., Williams, R. J., Werner, P. A., Bradshaw, C. J. A. & Bowman, D. M. J. S. 2006 Environmental and allometric drivers of tree growth rates in a north Australian savanna. *Forest Ecology and Management* **234**, 164-180. (DOI:10.1016/j.foreco.2006.06.034).

[119] Woinarski, J. C. Z., Armstrong, M., Brennan, K., Fisher, A., Griffiths, A. D., Hill, B., Milne, D. J., Palmer, C., Ward, S., Watson, M., et al. 2010 Monitoring indicates rapid and severe decline of native small mammals in Kakadu National Park, northern Australia. *Wildlife Research* **37**, 116-126. (DOI:10.1071/wr09125).

[120] Lawes, M. J., Murphy, B. P., Fisher, A., Woinarski, J. C. Z., Edwards, A. C. & Russell-Smith, J. 2015 Small mammals decline with increasing fire extent in northern Australia: evidence from long-term monitoring in Kakadu National Park. *International Journal of Wildland Fire* **24**, 712-722.

[121] Robinson, C. J. & Whitehead, P. 2003 Cross-cultural management of pest animal damage: A case study of feral Buffalo control in Australia's Kakadu National Park. *Environmental Management* **32**, 445-458. (DOI:10.1007/s00267-003-0013-6).

[122] Russell-Smith, J., Evans, J., Edwards, A. C. & Simms, A. 2017 Assessing ecological performance thresholds in fire-prone Kakadu National Park, northern Australia. *Ecosphere* **8**, Article e01856 (DOI:10.1002/ecs2.1856).

[123] Bell, R. H. V. 1982 The effect of soil nutrient availability on community structure in African ecosystems. In *Ecology of Tropical Savannas* (eds. B. J. Huntley & B. H. Walker). New York, Springer-Verlag.

[124] Scholes, R. J. & Walker, B. H. 1993 *An African savanna: a synthesis of the Nylsvlei study*. Cambridge, Cambridge University Press.

[125] Archibald, S. & Hempson, G. P. 2016 Competing consumers: contrasting the patterns and impacts of fire and mammalian herbivory in Africa. *Philosophical Transactions of the Royal Society B: Biological Sciences* **371**.

[126] Bond William, J., Smythe, K. A. & Balfour Dave, A. 2008 Acacia species turnover in space and time in an African savanna. *Journal of Biogeography* **28**, 117-128. (DOI:10.1046/j.1365-2699.2001.00506.x).

[127] Le Roux, E., Clinning, G., Druce, D. J., Owen-Smith, N., Graf, J. & Cromsigt, J. P. G. M. 2017 Temporal Changes in the Large Herbivore Fauna of Hluhluwe-iMfolozi Park. In *Conserving Africa’s Mega-Diversity in the Anthropocene: The Hluhluwe-iMfolozi Park Story* (eds. J. P. G. M. Cromsigt, S. Archibald & N. O. S. Owen-Smith), p. 80. Cambridge, Cambridge University Press.

[128] Staver, A. C., Bond William, J., Cramer Michael, D. & Wakeling Julia, L. 2012 Top‐down determinants of niche structure and adaptation among African Acacias. *Ecology Letters* **15**, 673-679. (DOI:10.1111/j.1461-0248.2012.01784.x).

[129] Cockcroft, M. J., Wilkinson, M. J. & Tyson, P. D. 1987 The application of a present-day climatic model to the late quaternary in southern Africa. *Climatic Change* **10**, 161-181. (DOI:10.1007/BF00140253).

[130] Archibald, S., Beckett, H., Bond, W. J., Ceotsee, C., Druce, D. J. & Staver, A. C. 2017 Interactions between Fire and Ecosystem Processes. In *Conserving Africa’s mega-diversity in the Anthropocene: the Hluhluwe iMfolozi Park story* (eds. J. P. G. M. Cromsigt, S. Archibald & N. Owen-Smith), pp. 233-264. Cambridge, Cambridge University Press.

[131] Walker, B. H., Emslie, R. H., Owen-Smith, R. N. & Scholes, R. J. 1987 To Cull or Not to Cull: Lessons from a Southern African Drought. *Journal of Applied Ecology* **24**, 381-401. (DOI:10.2307/2403882).

[132] Archibald, S. 2008 African Grazing Lawns—How Fire, Rainfall, and Grazer Numbers Interact to Affect Grass Community States. *Journal of Wildlife Management* **72**, 492-501. (DOI:10.2193/2007-045).

[133] Waldram, M., Bond, W. & Stock, W. 2008 Ecological engineering by a mega-grazer: white rhino impacts on a South African savanna. *Ecosystems* **11**, 101-112.

[134] Hempson, G. P., Archibald, S. & Bond, W. J. 2015 A continent-wide assessment of the form and intensity of large mammal herbivory in Africa. *Science* **350**, 1056-1061.

[135] Cromsigt Joris, P. G. M. & Beest, M. 2014 Restoration of a megaherbivore: landscape‐level impacts of white rhinoceros in Kruger National Park, South Africa. *Journal of Ecology* **102**, 566-575. (DOI:10.1111/1365-2745.12218).
